# Quantifying the effects of pseudonymisation on epidemiological research reliability: a tailored evaluation using a clinical data warehouse

**DOI:** 10.1186/s12911-026-03360-0

**Published:** 2026-02-19

**Authors:** Ariel Cohen, Yannick Jacob, Gilles Chatellier, Charline Jean, Benoît Playe, Alexandre Mouchet, Etienne Audureau, Antoine Boutet, Romain Bey

**Affiliations:** 1 Innovation and Data Unit, IT Department, Assistance Publique-Hôpitaux de Paris, Paris, France; 2 LIMICS, INSERM, Sorbonne Université, Paris, France; 3 Université Paris-Est Créteil, INSERM, IMRB U955, Créteil, France; 4 Clinical Research Unit, Department of Public Health, Assistance Publique Hôpitaux de Paris, Henri Mondor and Albert Chenevier Teaching Hospital, Créteil, France; 5 INSA Lyon, Inria, CITI, UR3720, Villeurbanne, 69621 France

**Keywords:** Electronic health record, Clinical data warehouse, Privacy, Pseudonymisation, Minimisation, Re-identification

## Abstract

**Background:**

Electronic health records (EHRs) hold immense potential for advancing medical research, but protecting patient privacy remains a critical challenge. Consequently, the choice of privacy-enhancing techniques must take into account the downstream analyses to preserve relevant data properties, often resulting in a trade-off between data utility and privacy. We aimed to evaluate different pseudonymisation algorithms and their impact in the context of six representative archetypal electronic health record epidemiological studies. This work seeks to empower Clinical Data Warehouse (CDW) stakeholders to make informed decisions that minimise privacy risks while ensuring information utility.

**Methods:**

We simulated various re-identification attempts conducted by an attacker with legitimate access to cohorts contained in the CDW of the Greater Paris University Hospitals. The dataset comprised 3,950,145 hospitalisation records with an admission between August 1st, 2017 and April 1st, 2024. We considered minimisation and pseudonymisation schemes with different parameterisations, randomly shifting the timestamps of the delivered data while preserving different degrees of temporal coherence among them. The impact of these techniques was assessed both on reliability of six representative archetypal epidemiological studies and on records uniqueness. Two attack scenarios were considered: a random-target attack and a target-in-cohort attack. Advantages and limitations of the different schemes were compared according to the specific requirements of the considered studies.

**Results:**

Attack success rates varied widely – ranging from a median of 0.9% [IQR: 0.3%-9.4%] in the random-target scenario to 99% [IQR: 86%-100%] in the target-in-cohort scenario – with minimisation accounting for most of this variability. Although less effective, pseudonymisation provided an additional reduction in re-identification risk. However, achieving low uniqueness required substantial modifications to temporal coherence, compromising the reliability of certain epidemiological statistics.

**Conclusions:**

Pseudonymisation must therefore be combined with other solutions, in particular data minimisation, to provide optimal privacy protection within CDWs. Our findings highlight the need for tailored data protection strategies that align with specific study objectives to preserve data utility for epidemiological research. Our findings will help Institutional Review Boards and CDW governance bodies and teams in making informed decisions to mitigate privacy risks while maintaining information utility.

**Supplementary Information:**

The online version contains supplementary material available at 10.1186/s12911-026-03360-0.

## Introduction

The digitisation of healthcare leads to the collection of increasing amounts of personally identifiable information, such as diagnoses, medications, clinical notes, and imaging tests. These data offer considerable opportunities for research, leading to the emergence of Clinical Data Warehouses (CDW) designed to facilitate their secondary use [1, 2]. The centralisation and analysis of millions of electronic health records (EHR) represents a challenge in terms of patients’ privacy protection, and various actions are consequently undertaken to limit the risk of patient re-identification. In particular, pseudonymisation enables making individuals less distinguishable by hiding them in numerous similar records, while data minimisation aims to reduce the amount of personal data delivered according to the final objective.

Pseudonymisation involves both removing directly identifying information (e.g., names, places of residence, etc.) [3] and blurring out data (i.e., generalising data) that could be used as indirect identifiers (e.g., providing one’s date of birth with precision at year rather than day level). Many indicators have been proposed to formally quantify the protection provided by pseudonymisation, among them uniqueness, i.e., the proportion of records that are unique in the database [4]. However, such pseudonymised records usually cannot be considered anonymous as an individual can often be singled out with little a priori knowledge about him or her [5, 6]. This limitation is observed in all studies involving human populations, including health [7, 8], DNA [9, 10], or mobility [11] to name a few. Hence, protecting patients’ privacy cannot rely solely on data pseudonymisation and it appears in particular necessary to combine it with a minimisation strategy of the data accessed by users or investigators, i.e., limit access to only those data directly relevant to the final objective.

In the context of a CDW, the implementation of pseudonymisation and its combination with minimisation poses particular challenges. On the one hand, a data user (e.g., physician, investigator, data scientist, etc.) with legitimate access to the cohorts contained in the CDW could use his or her a priori knowledge to single out and re-identify patients. On the other hand, as pseudonymisation modifies data, it may affect the reliability of downstream analyses. Depending on its scheme and parameterisation, a pseudonymisation algorithm can be either harmless or detrimental to a downstream analysis. It is therefore necessary to develop pseudonymisation algorithms that are adapted to the specificities of EHR-based studies [12–16].

In this study, we evaluate various pseudonymisation and minimisation schemes in the context of six archetypal EHR-based studies conducted on either small or large cohorts contained in the CDW of the Greater Paris University Hospitals (Assistance Publique-Hôpitaux de Paris, AP-HP). We quantify the impact of these schemes on data privacy and on the reliability of the EHR-based studies. Our intention is not to propose a novel de-identification method nor to provide a systematic comparison across techniques, but rather to assess how different commonly used date shifting techniques affects epidemiological reliability. We aim that our findings will help Institutional Review Boards (IRBs) and CDW governance and teams to make better decisions to minimise privacy risks, while ensuring the utility of the information.

## Methods

### Study design and population

This retrospective cohort study comprises patients with at least one hospital admission between August 1st, 2017 and April 1st 2024. Each patient’s record contains demographic data (sex, date of birth, date of death), administrative data relative to hospitalisations (admission dates, discharge dates, hospitals) and claim ICD-10 codes. All these records were collected on a daily routine using the AP-HP EHR software and accessed via a standardised database in the Observational Medical Outcomes Partnership (OMOP) format.

AP-HP comprises 38 hospitals spread across the Paris region (22,000 beds, 1.3 million hospitalisations per year). Analysis was conducted on April 2nd, 2024. We discarded incomplete records.

This research was approved by our institutional review board (IRB00011591), decision CSE23-13_EDS-PRIVACY.

### Data utility for epidemiological research

CDW can be used to conduct a wide variety of epidemiological studies. In this article we considered six archetypal scenarios consisting in retrospective observational cohort studies (inspired by published research using secondary data) [17–21]. We evaluated the impact of pseudonymisation and minimisation on their results through different plots or data descriptors illustrating thus the reduction of data utility for epidemiological research, i.e., the extent to which the pseudonymised data allows required analysis to be made. We moreover defined for each scenario a unique unreliability indicator. This indicator quantifies the extent to which the results of an epidemiological study conducted on pseudonymised data deviate from those obtained using the original dataset. For each use case, clinically meaningful study variables were identified (e.g., mean age at admission, proportion of three-month re-hospitalisations). The indicator takes positive values when these variables differ from the corresponding values in the non-pseudonymised data and—except in epidemic detection studies (bronchiolitis and influenza)—can be interpreted as the proportion of variation relative to the original result. For example, an unreliability score of 0.373 in the hospitalisation characterisation study indicates that key variables (e.g., age at admission, re-hospitalisation rate) deviate on average by 37.3% compared with the original dataset.

We simulated the following epidemiological research scenarios (see Supplement - section I for details on cohorts and indicators):

- **Characterisation of all hospitalisations.** We considered the overall cohort of inpatients. We measured the patients average age and standard deviation at first admission, and the proportion of three-month re-hospitalisation following discharge.
- **Seasonal bronchiolitis epidemic** [17]. We measured the weekly number of hospitalisations related to bronchiolitis in the study period and the average age at admission. We did not use a parametric model to fit this time series, but we compared the pseudonymisation-induced variations using Kullback-Leibler divergence^1^ and plotting the weekly number of hospitalisations.
- **Seasonal flu epidemic** [18]. We applied the same methodology as in the bronchiolitis scenario. We chose this second epidemic because it affects older patients and the age indicator should consequently be proportionally less sensitive to the pseudonymisation of the date of birth.
- **Readmission after bariatric surgery** [19]. We considered hospitalisations for bariatric surgery and we measured the 30-day hospital readmission rate.
- **Survival of pancreatic cancer patients** [20]. We considered new cases of pancreatic cancer. We plotted Kaplan-Meier curves to show differences between pseudonymisation algorithms. We computed utility variations measuring the results of a multivariate Cox regression model including sex, age at first diagnosis and treatment categories (surgery, chemotherapy, exclusive best supportive care).
- **Care pathways of cancer patients** [21]. We considered hospitalisations related to cancer and mimicked an analysis aiming at detecting common patterns among care pathways. For each patient, we computed a similarity distance (CHI-2) between hospitalisation sequences [22] and birth date. We applied a k-medoid clustering algorithm to group similar sequences and detect similarities among these high-dimensional data. We described each group using variables related to the utilisation of the healthcare system. This research scenario is introduced to represent emerging study designs that leverage the opportunities offered by high dimensional datasets. See supplement - section I and e-Fig. 1 for details.

### Pseudonymisation and minimisation

**Fig. 1:**
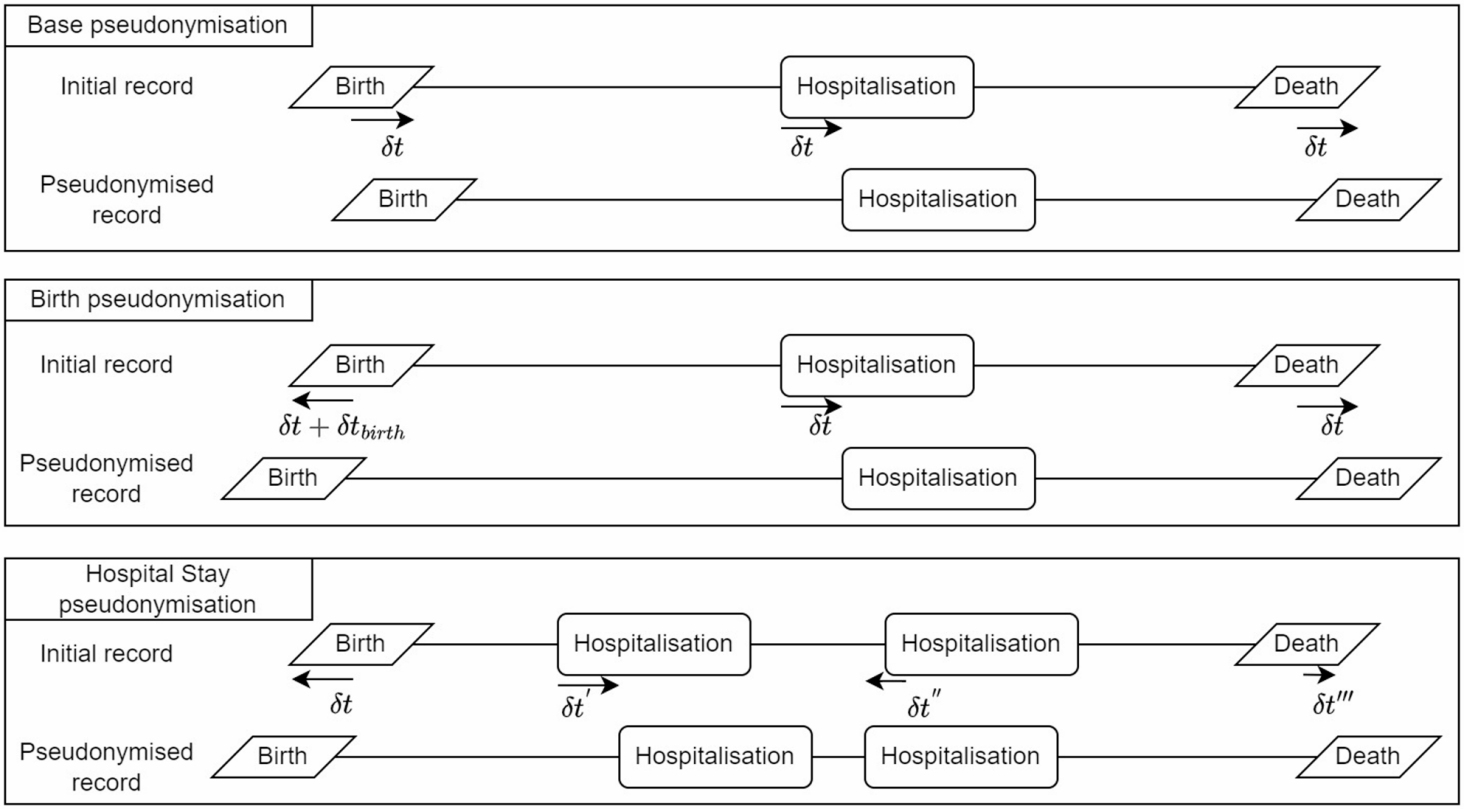
Three different pseudonymisation schemes used to enhance privacy by modifying dates. From top to bottom, the per-patient temporal coherence of data is increasingly altered to lower the average uniqueness of records

We evaluated three pseudonymisation schemes based on date shifting with different impacts on the temporal coherence of data at the patient level (see Fig. 1). Date shifting (with a unique random seed for each patient) is considered a form of generalisation, as the attacker could infer only a range of the real date. The three schemes are the following:

- **Base pseudonymisation**: all the event dates $\:t$ were replaced by $\:t+\delta\:t$, with $\:\delta\:t$ a per-patient variable that has been drawn in a uniform distribution $\:[-\varDelta\:t;+\varDelta\:t]$.
- **Birth pseudonymisation**: after having applied the *Base pseudonymisation* scheme, the date of birth was additionally shifted by a value $\:\delta\:{t}_{birth}$ drawn per-patient in a uniform distribution $\:[-\varDelta\:{t}_{birth};+\varDelta\:{t}_{birth}]$.
- **Hospital stay pseudonymisation**: The date of birth, date of death and stay dates (i.e., admission and discharge dates) were shifted by a different $\:\delta\:t$ value drawn uniformly in $\:[-\varDelta\:t;+\varDelta\:t]$. Admission and discharge dates were shifted with the same $\:\delta\:t$ value, so length of stay was conserved.

A pseudonymisation algorithm is the combination of one of these three schemes and a parameterisation (i.e., setting a value of $\:\varDelta\:t$).

We note that each scheme provides a distinct balance between privacy and temporal coherence. The Base scheme applies a single constant offset per patient across all dates, thereby preserving intra-patient intervals (e.g., time-to-event, time-to-readmission). The Birth scheme introduces a second offset for the date of birth, improving privacy by dissociating age from clinical events but slightly altering age-dependent analysis. Finally, the Hospital Stay scheme independently shifts birth, death, and stay periods, breaking temporal coherence and potentially reversing event order.

We evaluated a minimisation scheme based on cohort size, i.e., initial filtering aimed at reducing the number of individuals whose records are considered. Specifically, we evaluated attacks across cohorts containing different numbers of patients, typically ranging from 3,580 to 1,938,898 individuals. The cohort size has a direct relationship with the probability of success of the re-identification attack (i.e., if the target is not in the cohort, the attack fails).

### Attacks

Attacks were carried out by someone with legitimate access to a minimised and pseudonymised cohort or by an attacker that would have obtained an investigator’s access credentials (e-Figure 2A). We simulate two scenarios: (i) they aimed at re-identifying a randomly selected record in the CDW of the AP-HP (“*random-target*”) (n.b., this record may not be present in the accessed cohort as the attacker does not know a priori if the patient is in it); and (ii) they know that the target is in the cohort (“*target-in-cohort*”). We also assumed that the attacker knew the patient’s sex, date of birth, admission and discharge dates, date of death and hospitals visited. The attacker had full knowledge of the pseudonymisation scheme and its parameterisation (Kerckhoffs’ principle).

In the *random-target* scenario, due to its selection process, the targeted record is accessed by the attacker with a probability $\:P\left(access\right)=\:{n}_{cohort}/{n}_{total}$ where$\:\:{n}_{cohort}$ and $\:{n}_{total}$ are the number of patients in the cohort and in the total dataset, respectively. Beyond accessing the targeted record, for the attack to be successful, the attacker must also be able to (i) single out the record among the accessed cohort and (ii) be confident that the isolated record was the targeted one. We assumed that a necessary and sufficient condition therefore was that the targeted record was unique in the total dataset with respect to the attacker a priori knowledge. Indeed, as shown by Rocher et al. [23], an attacker can often infer with a high degree of confidence whether a record is unique in a total dataset even when accessing only a minimised subset. For the sake of simplicity, we did not explicitly simulate this complex inference and assumed that the attacker could perform it. If the record was unique in the total dataset, there was no uncertainty that a singled-out record in the cohort was the targeted one. On the contrary, in the *random-target* scenario, if a record of the cohort was not unique in the total dataset, singling it out in the minimised cohort cannot guarantee that it was the targeted one (because the attacker does not know a priori whether or not the targeted record is in the accessed dataset). This is not the case in the *target-in-cohort* scenario, where we checked if the target was unique in the accessed cohort instead of on the overall dataset (in this case the attacker knows that the targeted record is in the accessed dataset). See Supplement - section III for details on uniqueness computation.

More precisely, a record was considered unique if there was only one record in the pseudonymised dataset whose attributes fell within the range of potential pseudonymised values given the a priori knowledge of the attacker (i.e., a pseudonymised record that shared the same sex and visited hospitals than the targeted record and whose dates were compatible with the pseudonymisation scheme and its parameterisation, see e-Figure 2C and e-Figure 3). We chose to evaluate the unicity of the patient journey based on some of the more characteristic elements that define it. As the attacker could leverage other additional information to reidentify a patient, the reported uniqueness is a lower bound. Finally, with $\:P\left(unique\right|cohort)$, the probability of a record within the cohort to be unique (uniqueness), the probability of an attack to be successful can be written as follows:

1 $$\:P\left(success\right)=P\left(access\right)\times\:P\left(unique\right|cohort)$$

Note that if the attacker is certain that the target is in the cohort ($\:P\left(access\right)=1$), then the probability of success is equal to the probability of being unique within the cohort.

### Outcomes and statistical analysis

First, for each one of the study scenarios we simulated re-identification attacks and measured their success rates varying both the pseudonymisation and the minimisation schemes and its parameterisation (e.g., $\:\varDelta\:t$ in [0; 7; 10; 30; 100; 1000] days) and, in each case, the impact of pseudonymisation on data utility was measured using the unreliability indicator, data descriptors, and graphically illustrated with characteristic plots. The trade-off between privacy-enhancement and study reliability was discussed qualitatively. Second, we graphically compared the respective impacts of two privacy-enhancing techniques, pseudonymisation and minimisation, on the attack’s success rate. Third, we compared the average value of uniqueness between pseudonymisation schemes, parameterisations, cohorts and varying the attacker’s a priori knowledge. Fourth, to estimate the optimal parameterisation of the pseudonymisation algorithms we independently varied the value of $\:\varDelta\:{t}_{birth}$ and $\:\varDelta\:t$ in the birth pseudonymisation scheme and estimated uniqueness in each one of these cases. Finally, we analysed the k-anonymity distribution for each pseudonymisation algorithm.

## Results

A total of 444 patients were discarded as their records did not contain gender information. We therefore considered a total dataset composed of 3,950,145 hospitalisations related to 1,938,898 patients (46% of males), median age at first stay 39.7 years [IQR: 16.4–65.6] with 10% with an available date of death. The dataset has a heterogeneous distribution as regards hospitals, age at admission, length of stay and number of hospitalisations per patient (See supplement - section IV).

The effect of simulating different epidemiological analyses and re-identification attacks in the case of the six different EHR studies contrasting the unreliability indicator with the success rate is shown in Table 1. In the absence of both minimisation and pseudonymisation, 94% of patients’ records were re-identified. The median success rate was 0.9% [IQR: 0.3%-9.4%] for the *random-target* scenario and 99% [IQR: 86%-100%] for the *target-in-cohort* scenario. The median uniqueness was 96% [IQR 83%-100%] for the *random-target* scenario and for the *target-in-cohort* scenario the uniqueness corresponds to success rate. The unreliability indicator is shown and the green light shows the settings where there is no change in the results of the use case scenarios. Whereas attacks conducted accessing the total dataset, pseudonymised or not, led to a median re-identification success rate of 86% [IQR 68%-87%], attacks on minimised, disease-specific cohorts were two orders of magnitude lower (median 0.8%, [IQR: 0.3%-1.2%] for the *random-target* scenario. This was not the case for the *target-in-cohort* scenario (median 100%, [IQR: 99%-100%] for the disease-specific cohorts.

**Fig. 2:**
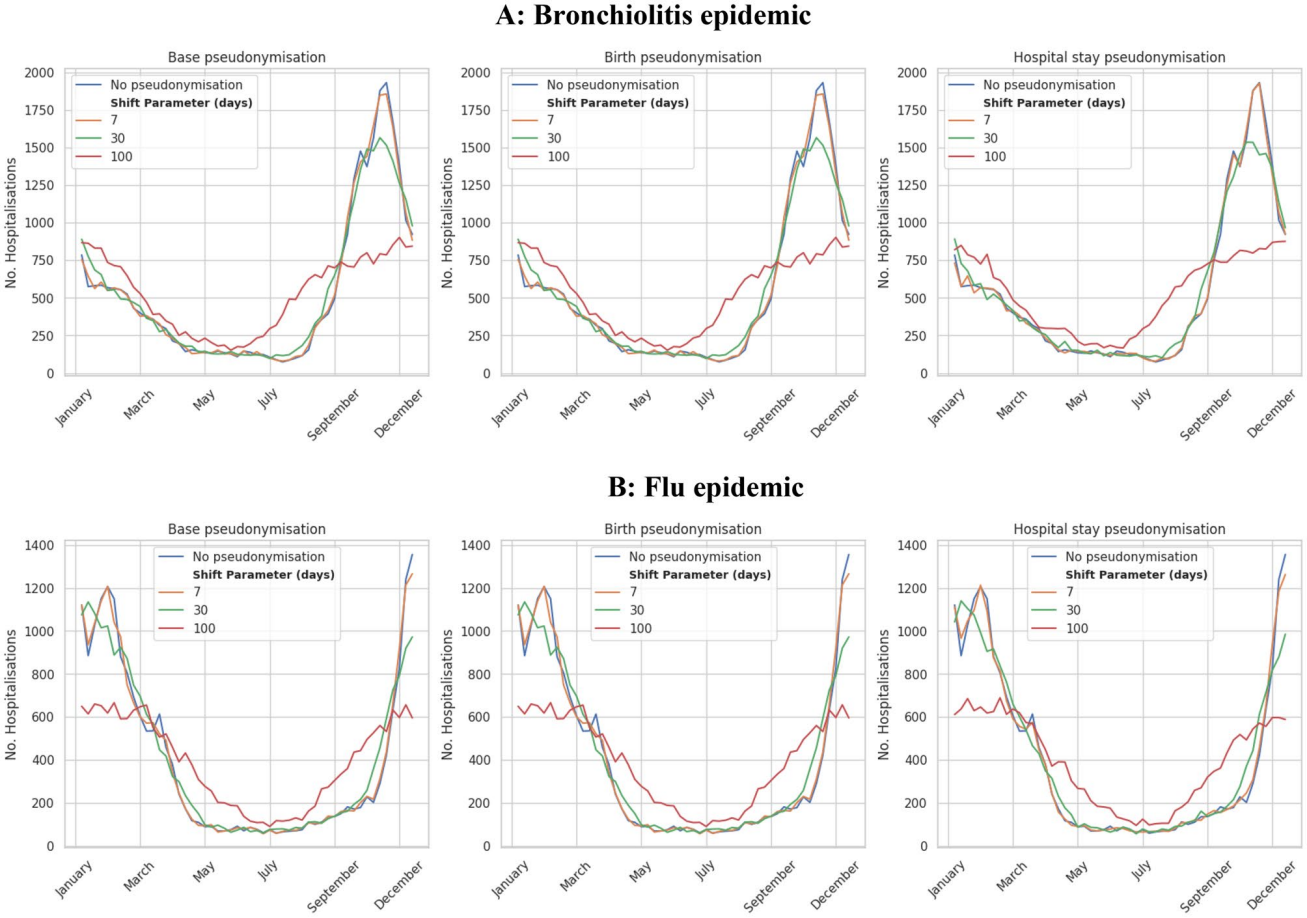
Influence of different pseudonymisation schemes and their parameterisation on the weekly number of hospitalisations over one year for bronchiolitis (panel **A**) or seasonal flu (panel **B**)

The effect of pseudonymisation on analytical utility of two study scenarios that consider seasonal epidemics is shown in Fig. 2 (weekly number of hospitalisations over one-year). The three proposed schemes significantly decreased the utility of data in a similar form beyond a 7-day shift, smoothing out the seasonality of both bronchiolitis and flu. As reported in Table 1, for date-shift amplitudes $\:\varDelta\:t\le\:7$ days, the unreliability indicator for both the bronchiolitis and seasonal influenza cohorts is ≤ 0.001, indicating an almost perfect preservation of weekly seasonality. This quantitative result confirms that small-amplitude shifts could preserve epidemiologically relevant temporal patterns.

**Fig. 3:**
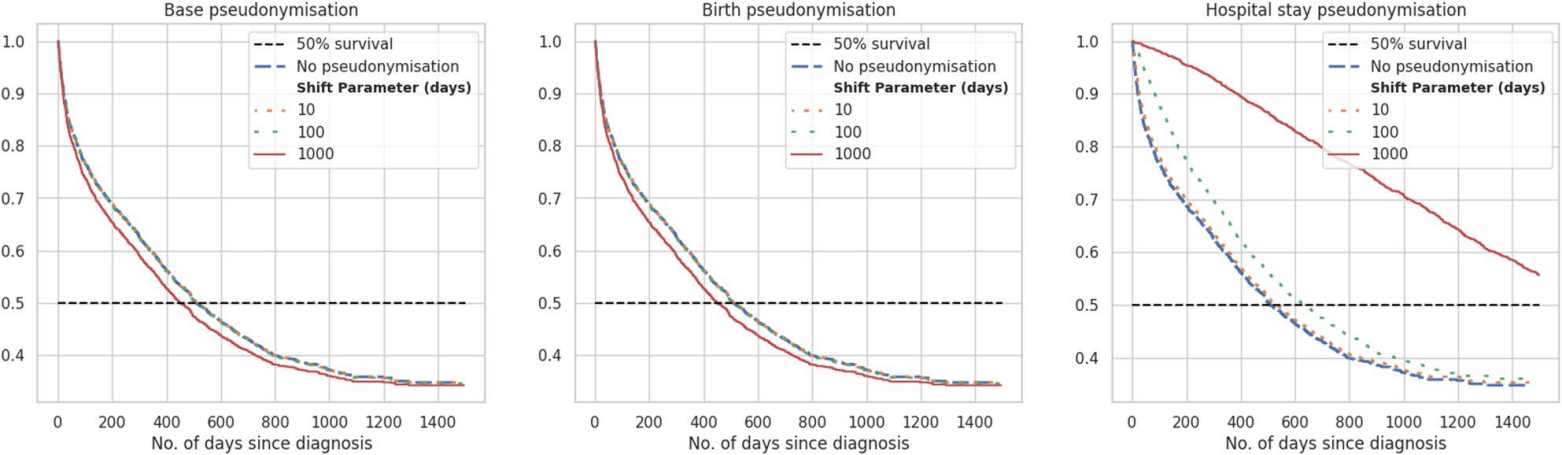
Effect of different pseudonymisation schemes, and their parameterisation on Kaplan-Meier survival curves in a cohort of patients with pancreatic cancer

In the case of the survival analysis of pancreatic cancer cases, Kaplan-Meier survival estimates for different pseudonymisation algorithms are shown on Fig. 3. The largest effect is observed with the stay-level scheme. The figure shows two mixed effects that affect the utility of the results: the distortion in time interval between diagnosis and death, and the non-inclusion of diagnosis or death events that are moved outside the study’s inclusion and follow-up dates by the pseudonymisation algorithm. Base and Birth pseudonymisation are found to be entirely suitable for this epidemiological research scenario, with parametrisations no greater than 100 days. For both schemes, the 10-day shift (orange) and 100-day shift (green) curves demonstrate a high degree of similarity to the original non-pseudonymised curve (blue), not modifying the clinical conclusions. Because the Stay scheme substantially distorts time-to-event intervals, we do not recommend its use in contexts where survival or longitudinal analyses are required.

**Fig. 4:**
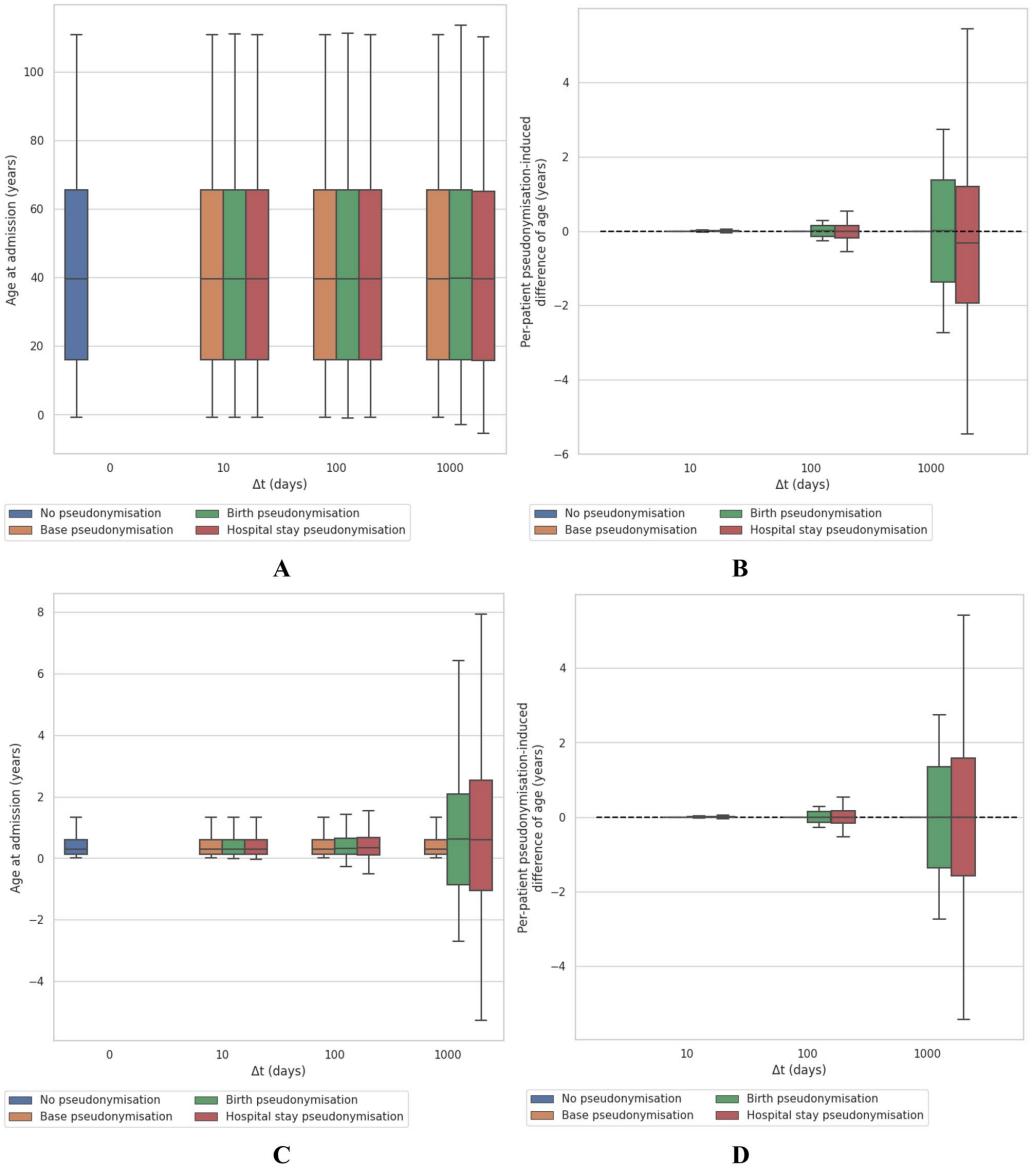
Boxplots of age at admission and per-patient difference of ages before and after pseudonymisation according to 3 pseudonymisation schemes parameterised with a 10-, 100-, and 1000-days shift. We considered the total cohort (panels **A** & **B**) and the bronchiolitis cohort (panels **C** & **D**)

The relationship between different pseudonymisation algorithms and a crucial aspect of data utility, age statistics, is shown on Fig. 4 for the total cohort and the bronchiolitis cohort. The impact on results is different for the two cohorts: the same parameterisation leads to different consequences depending on the mean age of the initial cohort. A shift parameter of 1,000 days using the birth or stay pseudonymisation scheme strongly affects medical findings for the bronchiolitis cohort, composed mainly of children. For the total cohort, when considering mean age at admission (left side of Fig. 4) and individual age differences (right side of Fig. 4) for each pseudonymisation algorithm we observe minor change in mean age at admission (i.e., data utility is conserved) despite an impairment of data fidelity at individual level. In certain situations, such as in stratified subgroup analysis, these perturbations can have a larger effect, especially if the size of the subgroups is limited.

In Table 2, we show different descriptors for each one of the four clusters using different pseudonymisation algorithms. For the Base pseudonymisation scheme there was no change in the cluster descriptors with respect to the absence of pseudonymisation, even with a shift parameter of 1,000 days. For the Birth and the Hospital stay pseudonymisation scheme, we obtained different cluster descriptors and memberships with respect to the absence of pseudonymisation.

**Fig. 5:**
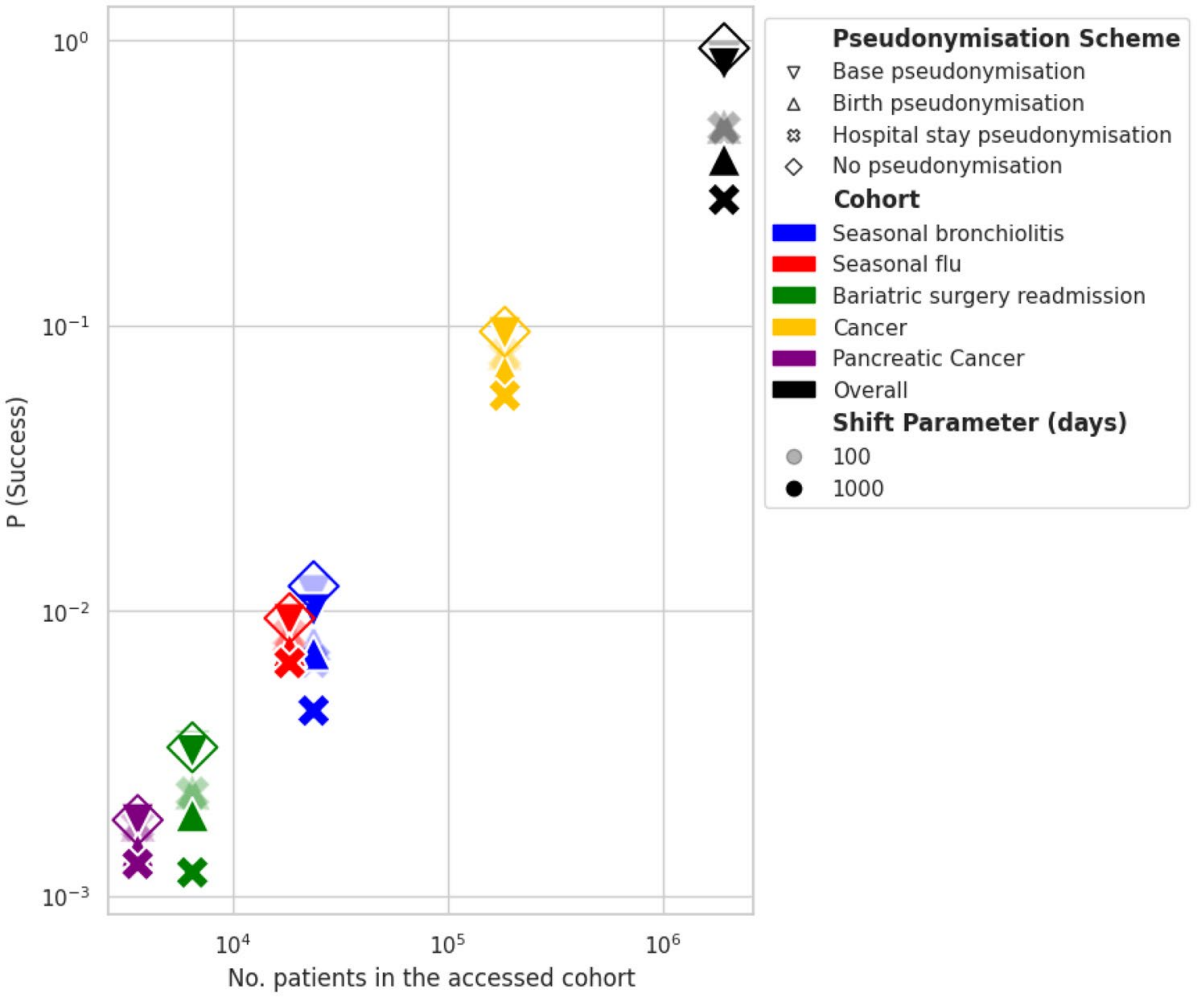
Impact of data minimisation and data pseudonymisation on the success rate of re-identification attacks. Colours stand for the different study scenarios and symbols for the pseudonymisation schemes and their parameterisation

**Fig. 6:**
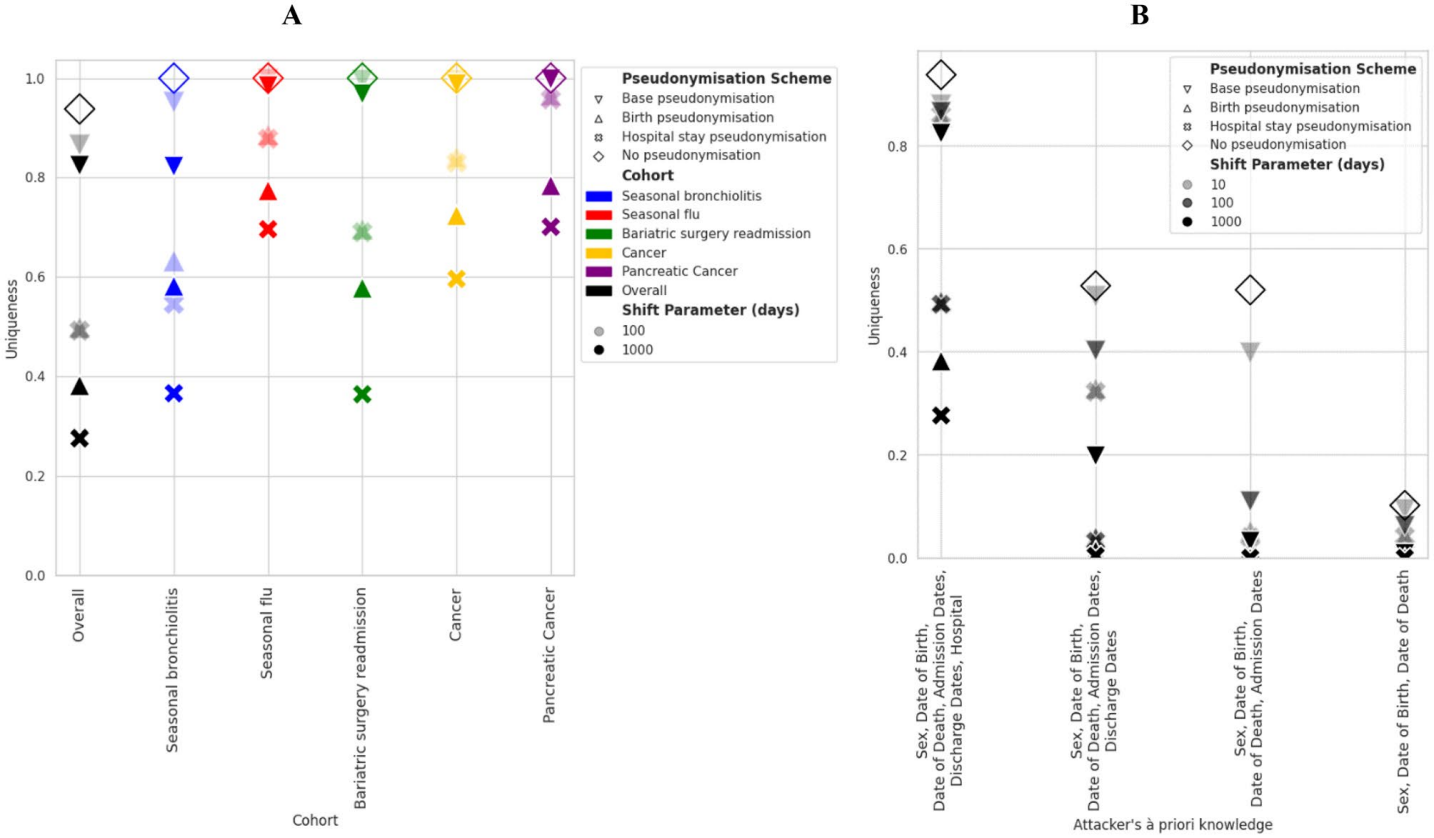
Variations of records’ average uniqueness (random-target scenario) with respect to **A**) population and **B**) the a priori knowledge of the attacker. For estimates shown in **B**) we considered the total cohort population

As shown on Fig. 5, most of the success rate variability of attacks in the *random-target* scenario was driven by the cohort size, i.e., by data minimisation. Figure 6a shows that uniqueness depends on both the pseudonymisation scheme and its parameterisation. Whereas the Base pseudonymisation scheme led to a small decrease of uniqueness, whatever its parameterisation, both Birth and Hospital Stay pseudonymisation schemes led to two- to four-fold decreases depending on their parameterisation. The more the temporal coherence between patients’ records was altered, the more uniqueness was lowered. For a given scheme, the greater the amplitude of the date shifting, the lower the uniqueness. The behaviour of uniqueness was loosely associated with the population under scrutiny, although both the flu and the cancer cohorts featured higher uniqueness when applying identical pseudonymisation schemes. Uniqueness was strongly associated with the a priori knowledge of the attacker (Fig. 6b) and with the proportion of records sharing the same attributes (hospital, patient’s age and sex, etc., see e-Figure 4).

**Fig. 7:**
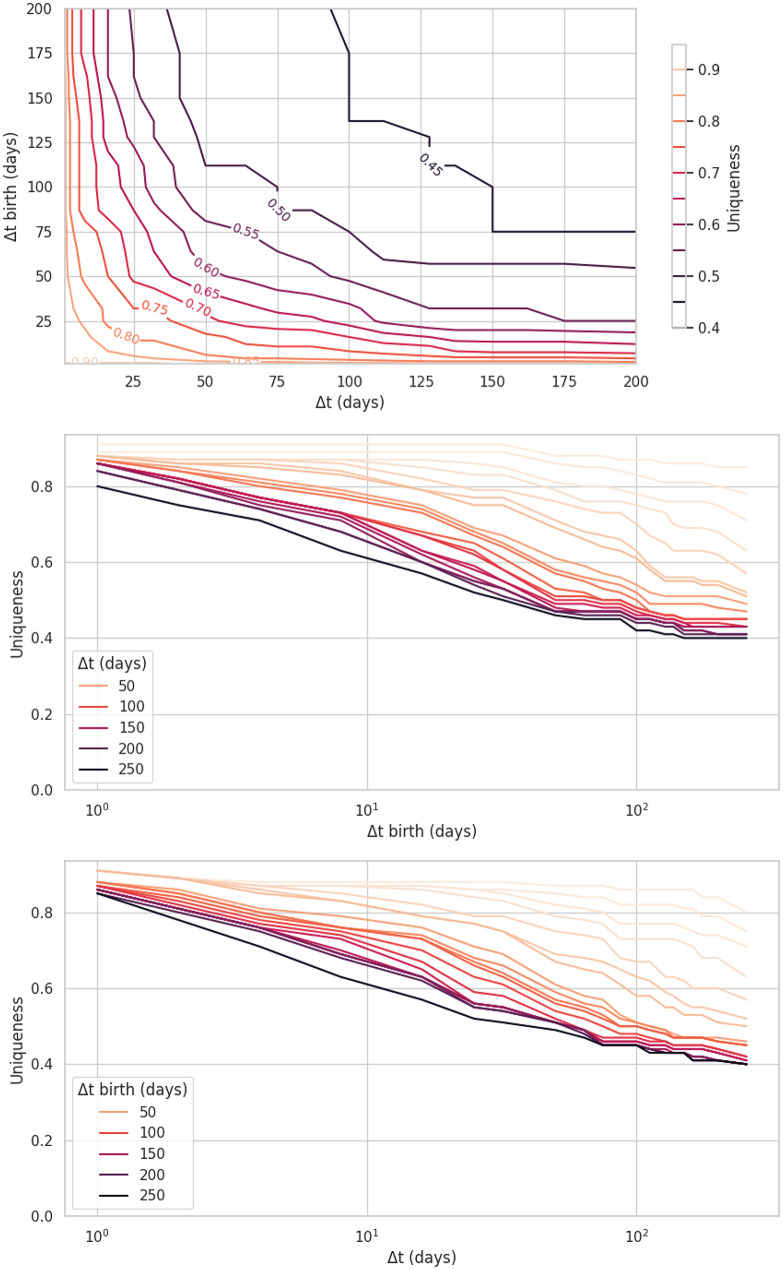
Comparison of uniqueness (random-target scenario) in the total cohort population applying the Birth Pseudonymisation scheme with different parameterisations: **A**) varying simultaneously the amplitudes of clinical events and birth shift parameters; **B**) and **C**) modifying only one parameter, the other one being kept constant

**Fig. 8:**
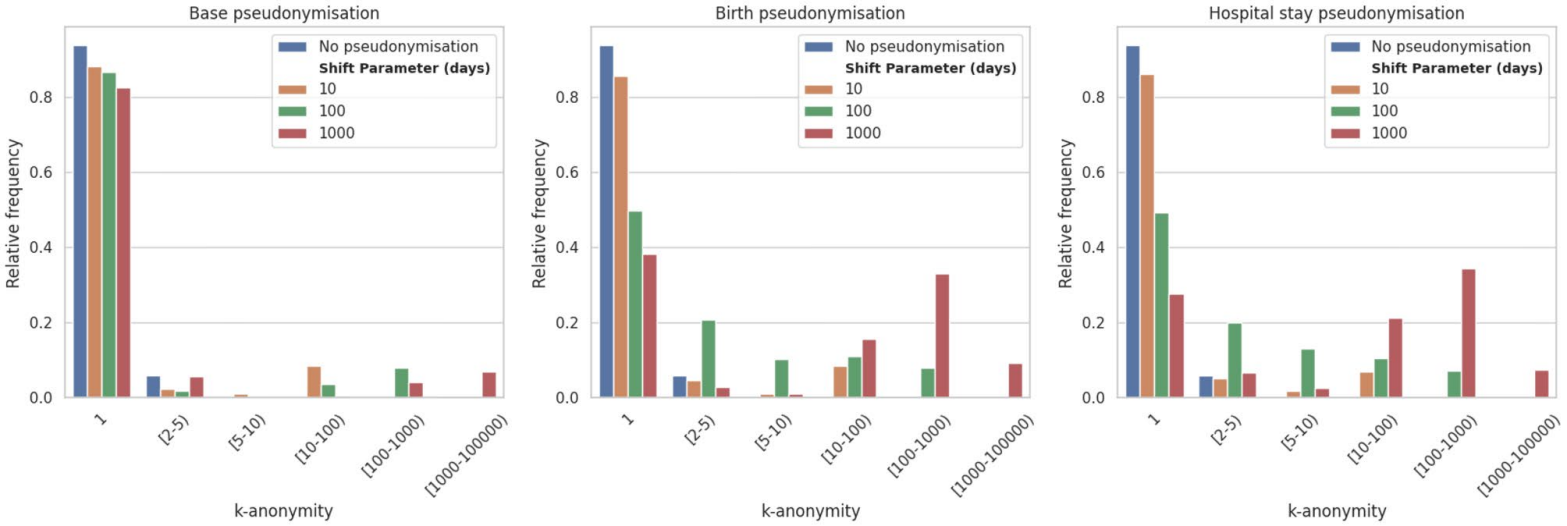
k-anonymity distribution for the overall cohort. The value of k = 1 correspond to the uniqueness

Focusing on a two-dimensional pseudonymisation scheme, here the Birth pseudonymisation scheme that consists in two per-patient temporal shifts $\:\delta\:t$ and $\:\delta\:{t}_{birth}$, an optimal parameterisation was obtained by applying modifications of comparable amplitudes on each temporal dimension simultaneously, i.e., the $\:\varDelta\:{t}_{birth}$ and $\:\varDelta\:t$ parameters (Fig. 7a). Acting on a single temporal dimension and keeping the other one fixed led to a logarithmically varying uniqueness (Fig. 7b and c). As well, Fig. 8 shows the k-anonymity distribution for each pseudonymisation algorithm, the Birth and Stay scheme provide more protection regarding this metric with respect to the Base scheme. We also observe similar results in k-anonymity distribution when comparing the Base and Hospital schemes. A k-suppression approach to achieve a k-anonymity ≥ 5 resulted in the suppression of more than 99% of records, when applied before pseudonymisation. When applied after pseudonymisation, record suppression remained substantial, with a median of 88% of records removed [IQR: 69%–90%], depending on the cohort and pseudonymisation scheme.

We observe in e-Table 1 the effects of age on both uniqueness and analytical utility. This table presents results stratified by age category for the overall cohort. We observed that both uniqueness and the unreliability indicator were consistently higher for the 90 + group compared with other age categories (29% higher than the population mean uniqueness; [IQR: 7–56%]. Conversely, the 0–14-year-old group was considerably less unique (54% lower than the population mean uniqueness; [IQR: 45–59%].

In e-Table 2 we present the detailed results for the *target-in-cohort* scenario and e-Table 3 describes the *random-target* scenario regarding the success rate.

**Table 1:**
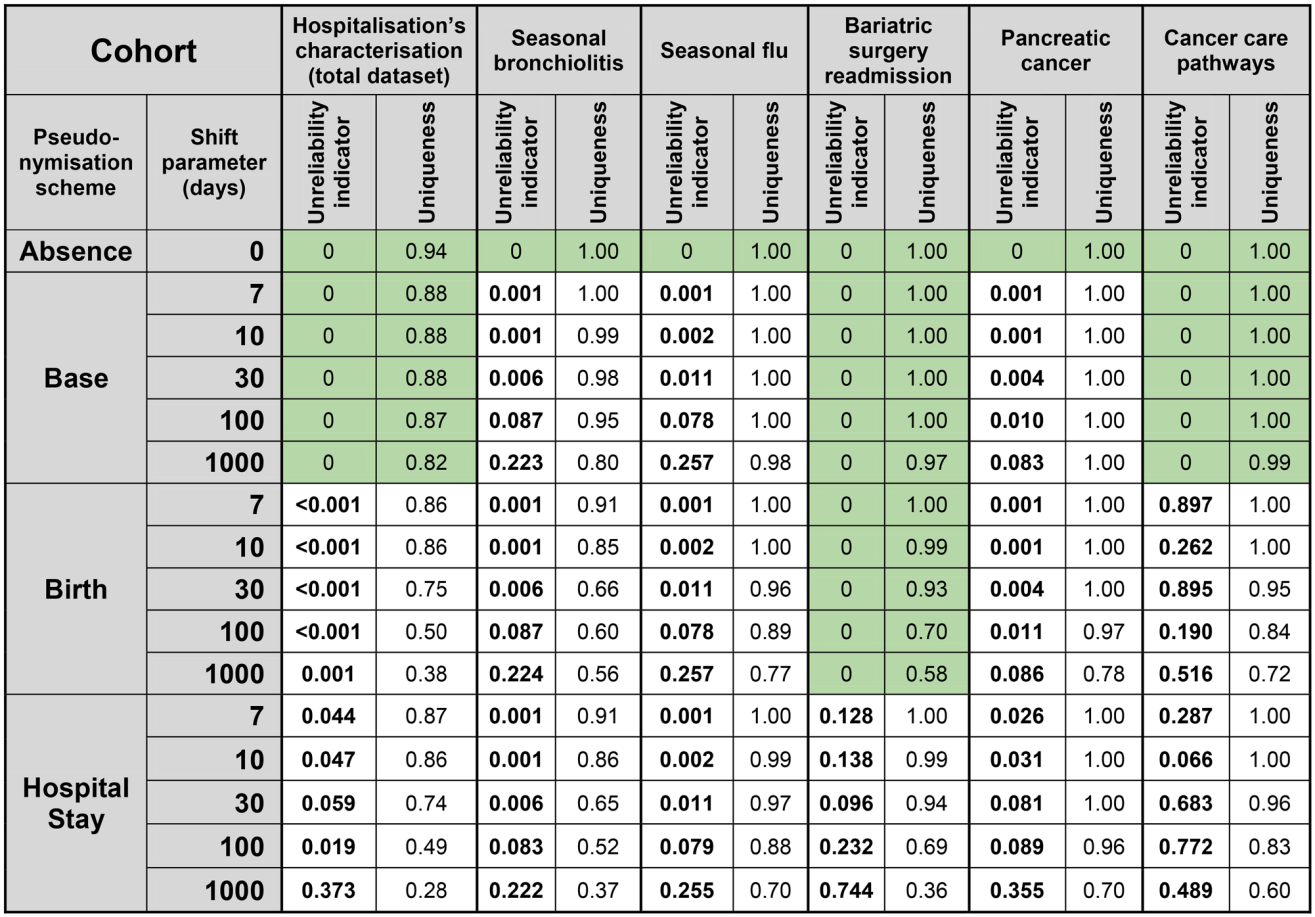
Association between privacy (random-target scenario) and unreliability of results estimated in the case of six archetypal electronic health record studies and three pseudonymisation schemes. Privacy was evaluated through the uniqueness in the dataset given attacker’s knowledge and integrity of a study’s result was evaluated through an unreliability indicator (see methods section), the lower the better in both cases. For each study we coloured in green the pseudonymisation schemes that did not impact reliability, i.e., no changes in the research results of each use case scenario. We highlight in bold Police the unreliability indicators that are impacted by the pseudonymisation algorithms vs. the absence of it

**Table 2:** Association between different pseudonymisation algorithms and descriptors of the clusters of cancer patients’ care pathways. We highlight in bold Police the descriptors that are impacted by the pseudonymisation algorithms vs. the absence of it

Pseudonymisation scheme (Shift parameter)	Number of patients in the cluster	Median time interval between stays (days)	Median stay duration (days)	Median number of stays	Median follow-up time (days)	Median age at admission
**Absence** **(0 day)**	562	74	6.0	1	27	68.6
	511	68	5.5	1	19	60.4
	498	80	6.3	1	32	76.7
	427	55	8.0	1	26	86.1
**Base** **(1000 days)**	562	74	6.0	1	27	68.6
	511	68	5.5	1	19	60.4
	498	80	6.3	1	32	76.7
	427	55	8.0	1	26	86.1
**Birth** **(1000 days)**	**783**	**69**	**6.0**	**1**	**22**	**61.4**
	**588**	**199**	**6.0**	**1**	**28**	**72.5**
	**409**	**129**	**8.0**	**1**	**32**	**82.8**
	**218**	**103**	**7.0**	**1**	**21**	**90.0**
**Hospital Stay** **(1000 days)**	**799**	**282**	**5.5**	**1**	**22**	**61.2**
	**448**	**308**	**6.7**	**2**	**90**	**71.7**
	**391**	**392**	**8.0**	**1**	**53**	**86.2**
	**360**	**237**	**6.5**	**1**	**63**	**78.9**

## Discussion

By applying different pseudonymisation and minimisation schemes in the context of six archetypal EHR studies, we demonstrated that such pre-processing should be adapted to each specific study design in order to ensure data utility for epidemiological research. On the one hand, pseudonymisation alters some properties of the data – in our case different temporal dimensions – and preserving the reliability of downstream analyses may therefore require disabling some of these modifications. On the other hand, if a statistical property of a dataset is not of interest for a specific study objective, altering it can enhance patient privacy without affecting the final results.

Our findings confirm that the choice of pseudonymisation scheme must depend on the intended analysis. We emphasise that some highly modifying schemes, such as stay pseudonymisation, may lead to spurious records, e.g., swapping the order of two hospitalisations. However, this artefact would not affect some analyses, for example when a study focuses on the sequence of events within a stay. On the contrary, as shown in Fig. 2, even the Base pseudonymisation scheme, the least altering, which preserves most of the temporal coherence of patient records, would not be appropriate (beyond certain parameter values) for studying seasonal epidemics, the effects of external events such as severe air pollution or the introduction of a medical product or procedure, since such studies require access to the exact dates of admission. As an alternative, seasonality-preserving pseudonymisation schemes could constrain date shifts to maintain the week-of-year alignment. This approach preserves weekly epidemic patterns while introducing uncertainty on absolute calendar dates or being of the form $\:\varDelta\:\:=k\:\times\:365+\:\epsilon\:$, particularly interesting for stationary epidemic settings. However, further research should be done on seasonality-preserving transformation schemes to understand their limits and to adapt them to non-stationary epidemics in order to avoid flattening or replicating epidemics waves across years.

Furthermore, for survival or time-to-event studies, maintaining intra-patient temporal coherence is crucial. Large or variable date shifts—especially those affecting death dates—can lead to spurious censoring or altered survival curves. Among the evaluated schemes in the survival of pancreatic cancer patient’s scenario, the Base and Birth schemes with parametrisations no greater than 100 days are found to be entirely suitable. Because the Stay scheme substantially distorts time-to-event intervals, we do not recommend its use in contexts where survival or longitudinal analyses are required.

To further evaluate how pseudonymisation may influence analytical validity beyond descriptive indicators, we examined its impact on regression-based survival modelling. Our evaluation of the Cox regression coefficients in the survival analysis of pancreatic cancer patients shows that moderate date-shifting amplitudes (≤ 100 days) preserve effect-size estimates almost perfectly—within approximately 1% distortion—under the Base and Birth schemes. In contrast, larger shifts or temporally incoherent schemes (e.g., Stay) markedly distort parameter estimates and can lead to unstable or attenuated effects. This evaluation framework can be readily extended to other regression or predictive models, providing a systematic means of assessing how pseudonymisation influences effect-size estimation, model stability, and variable selection.

Uniqueness was also not robust to variations in the attacker a priori knowledge, nor to cohort specificities or statistical outliers (i.e., records containing values rarely observed). Moreover, uniqueness varied across age groups (e-Table 1): older patients (≥ 90 years) exhibited more distinctive care pathways than the rest of the population, whereas children (0–14 years) had more homogeneous records, allowing effective protection with minimal data perturbation. As these parameters are loosely controlled in real-life contexts, when considered by IRB members, the uniqueness measured in our study should be considered as lower bounds of actual risks, a real attack could also leverage additional information. The aim of our study is to focus on the unicity of the patient journey and evaluate some of the more characteristic elements that define it (admission and discharge dates and place of service).

Our results are in qualitative accordance with the findings of Montjoye et al. [4, 11], particularly the logarithmic variation of uniqueness when varying just a single parameter of the pseudonymisation scheme (Fig. 7), the gain in terms of protection is greater when relatively small perturbations are applied jointly to several variables. More generally, our results confirm previous findings showing altered epidemiological results due to pseudonymisation [12, 13]. Our analyses also confirm that the results of studies that detect high-dimensional patterns, using for instance clustering algorithms, are difficult to preserve when applying pseudonymisation [12, 24].

Although pseudonymisation provided a measurable privacy gain, its effect was, on average, substantially lower than that achieved through data minimisation (Figs. 5 and 6). In line with this observation, the *target-in-cohort* scenario demonstrates that reducing an attacker’s certainty regarding whether a target is included in the accessed cohort (P(access)) is crucial, particularly given the high levels of uniqueness observed when the attacker is certain of the patient’s presence. A straightforward approach to data minimisation is to randomly sample a subpopulation of the cohort of interest. For example, if an investigator conducts research on a cohort of pancreatic cancer patients defined by specific inclusion and exclusion criteria, Clinical Data Warehouse (CDW) custodians could provide a random sample of that cohort to reduce the attacker’s certainty about P(access). In this situation, even if the attacker knows that the targeted patient meets the cohort’s criteria, they cannot be certain that the patient is actually included in the dataset.

Date shifting is widely employed in the context of secondary use of clinical data and is often considered a sufficient pseudonymisation technique from a legal point of view to give data access for secondary use. Even though, pseudonymisation must therefore be combined with other solutions, in particular data minimisation, to optimally protect privacy in the context of CDWs. When minimisation may itself alter study results, as it may for instance be the case in exploratory analyses or when training some artificial intelligence algorithms, other privacy-enhancing solutions such as adapted workflows may be introduced [25].

Interpreting success rates and uniqueness metrics in practical terms is crucial, particularly for decision- and policy-makers overseeing Clinical Data Warehouses. Under the General Data Protection Regulation (GDPR), data qualify as anonymous only if they prevent (i) singling out, (ii) linkability, and (iii) inference of personal attributes. The uniqueness and k-anonymity metrics are directly linked to the singling-out requirement. However, because neither the GDPR nor the French Data Protection Authority (CNIL) define quantitative thresholds for a “very low risk” level of anonymisation, interpreting these metrics remains challenging. The k-anonymity distribution shown in Fig. 8 illustrates the difficulty of achieving a substantial proportion of patients with k-anonymity values greater or equal than 5. The results of a k-suppression approach indicate that only a very small fraction of the dataset could be retained while satisfying the k-anonymity constraint. This extensive data loss renders the datasets unsuitable for meaningful epidemiological analyses, highlighting the practical limitations of k-suppression when applied to rich longitudinal EHR data. This finding also suggests that uniqueness (i.e., cases where k = 1) can serve as a practical scalar indicator of protection against singling out and may therefore inform data protection decisions.

Our study presents several limitations. First, we have simulated attacks exploiting only a few categories of data. In a real attack, it would be possible to single out records using additional information related to biological tests, comorbidities, etc. Furthermore, in the *random-target* scenario, we assumed that the targeted record was randomly selected from the total cohort, whereas in real life an attacker could also opportunistically target one that is in his/her cohort. Second, privacy-leakage is not limited to re-identification as an attacker may also infer health information or link records in different cohorts. We did not consider more advanced privacy technologies such as differential privacy, which are well known to degrade utility, nor other minimisation schemes such as variable selection. Third, we did not assess the impact for patients of successful re-identification although this aspect is crucial to estimate a dataset sensitivity. For instance, certain categories of data such as genomic data or clinical notes may be especially sensitive as they also expose privacy of the patient’s relatives [26, 27]. Estimating a dataset sensitivity therefore requires, beyond purely statistical approaches, taking into account the opinion of various stakeholders, in particular patients [28]. Fourth, we have only superficially explored the problem of pseudonymisation of high-dimensional data. Nevertheless, our clustering study could easily be extended to investigate the impact of pseudonymisation on other clustering analyses. The need for such test-beds is growing as new pseudonymisation technologies have yet to be thoroughly evaluated on realistic datasets and on innovative research designs [29, 30].

Privacy is considered one of the essential characteristics of the doctor-patient relationship, as stipulated in the Hippocratic Oath. This paradigm is being challenged in the era of big data as more and more scientists are able to analyse health records of large populations. In this configuration, although pseudonymisation and minimisation may enhance privacy, there is always a residual exposure of patient privacy that depends on the design of information systems. Associating various stakeholders to their specification, including patients, clinicians and scientists, is therefore necessary to build a long-lasting trust. This work is a step in that direction, as it aims to reconcile critical assessment of privacy risks with the optimisation of pseudonymisation algorithms that lower that risk, while ensuring studies’ utility.

## Supplementary Information

Below is the link to the electronic supplementary material.

Supplementary Material 1

## Data Availability

Access to the clinical data warehouse’s raw data can be granted following the process described on its website: http://www.eds.aphp.fr. A prior validation of the access by the local institutional review board is required. In the case of non-AP-HP researchers, the signature of a collaboration contract is mandatory. The computer code developed to perform these analyses has been made freely available online: https://github.com/aphp-datascience/study-privacy.

## Abbreviations

EHR: Electronic health records
CDW: Clinical Data Warehouse
IQR: Interquartile range
IRBs: Institutional Review Boards
AP-HP: Assistance Publique-Hôpitaux de Paris
ICD10: International Classification of Diseases, 10th revision
OMOP: Observational Medical Outcomes Partnership

## Footnotes

1 Measure of how different two distributions are, with value 0 if and only if the two distributions in question are identical.
