# Supplementary material for: Quantifying the effects of pseudonymisation on epidemiological research reliability: a tailored evaluation using a clinical data warehouse
Source: BMC Med Inform Decis Mak. 2026 Feb 19;26:87. doi: 10.1186/s12911-026-03360-0 (PMC13020004; doi:10.1186/s12911-026-03360-0)
Supplement: Supplementary file 1 — Supplementary Material 1 [file 12911_2026_3360_MOESM1_ESM.pdf]

Supplementary Material

Quantifying the Effects of Pseudonymisation on Epidemiological Research Reliability: a Tailored Evaluation Using a Clinical Data Warehouse

List of contents

I. Details on archetypal EHR studies 1

A. Characterisation of all the hospitalisations 1

B. Seasonal bronchiolitis epidemic 1

C. Seasonal flu epidemic 1

D. Readmission after a bariatric surgery 1

E. Survival of pancreatic cancer patients 2

F. Care pathway of cancer patients 3

II. Roles and data flow on AP-HP's clinical data warehouse 5

III. Uniqueness computation 7

IV. Advanced description of the population 9

V. Target-in-cohort scenario 12

VI. Success rate – random-target scenario 13

VII. References 14

VIII. RECORD's statement checklist 15

List of figures

- *e-Figure1: Patients' care pathways representation for each cluster of cancer patients*
- *e-Figure2: Dataflows and processing*
- *e-Figure3: Comparison of records to compute uniqueness*
- *e-Figure4: Number of records and average uniqueness of various subgroups*

I. Details on archetypal EHR studies

In this section we provide additional information on the five archetypal use cases, including in particular the definition of inclusion criteria and the unreliability indicators.

A. Characterisation of all the hospitalisations

Inclusion criteria:
We considered patients with at least one hospital admission between August 1st, 2017 and April 1st, 2024.

Unreliability indicator:
The following statistics were computed: mean age at admission of the first hospitalisation and its variance (s_1 and s_2), and proportion of re-hospitalisation in the three months following discharge of this hospitalisation (s_3). The unreliability indicator was defined as $|{s_1}^p - {s_1}^0|/3{s_1}^0 + |{s_2}^p - {s_2}^0|/3{s_2}^0 + |{s_3}^p - {s_3}^0|/3{s_3}^0$ with p and 0 superscript standing for the values of the statistics after and before pseudonymisation, respectively.

B. Seasonal bronchiolitis epidemic

Inclusion criteria:
We considered patients with at least one hospital admission between August 1st, 2017 and March 9th, 2023, and we kept only hospital stays with at least one bronchiolitis diagnostic code (J210, J218, J219).

Unreliability indicator:
The unreliability indicator was defined as $|{s_1}^p - {s_1}^0|/2{s_1}^0 + KL(d^p||d^0)/2$ with KL the Kullback-Leibler divergence between the temporal distribution of cases after and before pseudonymisation. s_1 represents the average age at admission, and p and 0 superscript stand for the values of the statistics after and before pseudonymisation, respectively.

C. Seasonal flu epidemic

Inclusion criteria:
We considered patients with at least one hospital admission between August 1st, 2017 and March 9th, 2023, and we kept only hospital stays with at least one seasonal flu diagnostic code (J09, J100, J101, J108, J110, J111, J118, J129, I411, G051) with patients aged over 15 at admission.

Unreliability indicator:
We used the same unreliability indicator as for the seasonal bronchiolitis cohort.

D. Readmission after a bariatric surgery

Inclusion criteria:
We considered patients with at least one hospital admission between August 1st, 2017 and March 9th, 2023, at least 18-year-old at the start of the stay, with an overweight and obesity ICD-10 diagnostic code (E66*) as main diagnosis registered for these hospital stays and one of the following CCAM

(Classification Commune des Actes Médicaux, French medical classification for clinical procedures) medical act codes:

CCAM Code	CCAM Label
HFMC007	Gastroplasty using an adjustable perigastric ring for obesity, by laparoscopy
HFCA001	Gastric bypass with Y-shaped loop [Y-shaped gastric bypass] for obesity, by laparotomy
HFCC003	Gastric short circuit with Y-shaped loop [Y-shaped gastric bypass] for obesity, by laparoscopy
HFFA011	Sleeve gastrectomy for obesity, by laparotomy
HFFC018	Longitudinal gastrectomy [Sleeve gastrectomy] for obesity, by laparoscopy
HFMA009	Gastroplasty using an adjustable perigastric band for obesity, by laparotomy

Unreliability indicator:
The 30-day hospital readmission rate was computed (s_1) and the unreliability indicator was defined as $|{s_1}^p - {s_1}^0|/{s_1}^0$.

E. Survival of pancreatic cancer patients

Inclusion criteria:
We included patients newly referred between January 1st, 2019 and December 31th, 2022, with at least one hospitalisation and a pancreatic cancer CIM-10 code (C25* codes, recorded either as principal or related diagnosis). The date of the first diagnostic code was taken as the inclusion date. We excluded patients having another hospitalisation with a C25* code occurring in the two years preceding this date, patients having multiple cancers and patients having endocrine tumours (C254 code) and patients who were not treated within AP-HP hospitals.

Unreliability indicator:
For each patient we gathered the following explanatory covariates: age at inclusion, sex and first treatment category received. The latter as divided into

- Systemic anticancer therapy (chemotherapy ICD-10 code Z511)
- Best supportive care (ICD-10 code Z515).
- Surgery (i.e.) pancreatectomy identified by the following CCAM codes:

CCAM Code	CCAM Label

HGFA014	Excision of the major duodenal papilla by laparotomy
HNFA001	Pancreatic isthmectomy with restoration of continuity of the pancreatic duct, by laparotomy
HNFA002	Left pancreatectomy with conservation of the spleen, with pancreatojejunal or pancreaticojejunal anastomosis, by laparotomy
HNFA004	Total duodenopancreatectomy with splenectomy [Total splenopancreatectomy], by laparotomy
HNFA005	Pancreatic tumour removal by laparotomy
HNFA006	Total or subtotal pancreatectomy with preservation of the duodenum and splenectomy, by laparotomy
HNFA007	Cephalic duodenopancreatectomy, by laparotomy
HNFA008	Left pancreatectomy with conservation of the spleen, by laparotomy
HNFA010	Left pancreatectomy with splenectomy [Left splenopancreatectomy] with pancreatojejunal or pancreaticojejunal anastomosis, by laparotomy
HNFA011	Total or subtotal pancreatectomy with preservation of the duodenum, without splenectomy, by laparotomy
HNFA013	Left pancreatectomy with splenectomy [Left splenopancreatectomy], by laparotomy
HNFC001	Laparoscopic removal of a pancreatic tumour
HNFC002	Left pancreatectomy with splenectomy [Left splenopancreatectomy], by laparoscopy
HNFC028	Left pancreatectomy with conservation of the spleen, by laparoscopy

Outcome was survival at time t, defined as the duration defined as the duration between the date of inclusion and the end of the observation period, December 31th, 2022. We modelled the survival using a Cox regression model with coefficients relative to the covariates. We fitted two models, one on the unaltered data and the other one on the pseudonymised data.

The unreliability indicator was defined as $\sum_i |(\beta_i^p - \beta_i^0)/\beta_i^0|$, with β_i the coefficients of the regression, and p and 0 superscripts standing for the values of the parameters after and before pseudonymisation, respectively.

F. Care pathway of cancer patients

Inclusion criteria:

We considered patients with at least one hospital admission between August 1st, 2017 and March 9th, 2023. We have considered hospitalisations with registered cancer ICD-10 diagnostic code (C*) as a main, related or associated diagnostic.

Unreliability indicator:

To identify similar care trajectories, we focused on the timing of admission and discharge dates relative to patients' 60th years anniversary. We considered all hospitalisations with a cancer-related diagnosis code and the patients' 60th anniversary for a sample of 2000 patients. Each sequence started with the admission date of the first hospitalisation (or the anniversary if earlier) and duration was measured from this index date. The CHI-2 distance between sequences was then calculated. As presented by Studer et al., the CHI-2 distance is indeed appropriate to characterise the duration between events. The choice of the 60th year instead of the date of birth was made in order to have shorter sequences, applying a fixed offset for all patients.

A k-medoid clustering algorithm (Partitioning Around Medoids) was then applied on the distance matrices before and after pseudonymisation. The number of clusters was set to 4 after inspection of the dendrogram computed before applying the pseudonymisation.

The unreliability indicator was defined as one minus the proportion of cases who were in the largest cluster before pseudonymisation that are still in the largest cluster after pseudonymisation (s_1):

$$1 - s_1$$

We also described in Table 2 each group using variables related to the utilisation of the healthcare system. As there is no direct identifier between the clusters across the different experiments, we sorted them by the number of patients of each cluster. e-Figure1 illustrates patients' trajectories for each cluster, where we observe the effects of timing between groups.

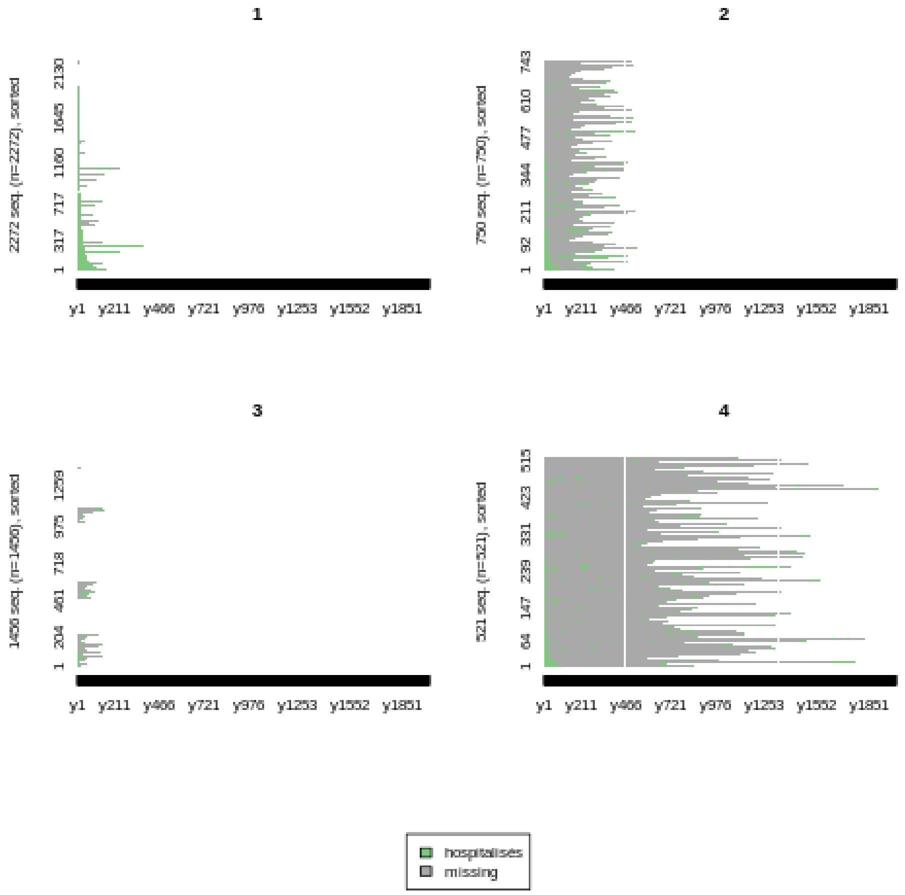

e-Figure 1: Cluster analysis of care pathways of cancer patients before applying any pseudonymisation. Patients' trajectories representation for each cluster

II. Roles and data flow on AP-HP's clinical data warehouse

Different roles are distinguished on AP-HP's CDW (see e-Figure2). Data collection and curation are performed by *clinicians* and *data custodians*, respectively. Curation includes a first stage where directly identifying information is suppressed, e.g., full names and places of residence (1). Once these tasks are completed, *data owners* include records of interest in study-specific cohorts of various sizes. A research protocol may then be submitted to the institutional review board (IRB) specifying the cohort and the categories of data whose access is requested by investigators. After IRB agreement (i.e., data are appropriate and minimized relatively to a legitimate research objective) the cohort's data are extracted from the total dataset and pseudonymised by generalisation consisting in modifying events' dates to lower the uniqueness of records. Different pseudonymisation schemes can be adopted and parameterized to optimally balance preserving statistical properties and enhancing privacy. Finally, data are delivered to *investigators* for analysis in study-specific secure environments.

Although minimisation appears as a cornerstone of privacy in the context of CDWs, it also features important drawbacks. In particular, it relies on a strict separation of roles as data selection is usually performed by data owners, not investigators, although this task is methodologically non-trivial and may induce biases (see e-Figure2A). Moreover, the very principle of minimisation may appear somehow incompatible with emerging study designs such as the training of machine learning models on large-scale databases (2). For these studies, a workaround may consist in a further decomposition of tasks and roles, distinguishing for instance an exploratory stage that could be realized on a minimized cohort from the large-scale training of the model on the total database. Whereas the former stage may be conducted by investigators, the latter may for instance be delegated to a small team of authorized machine learning engineers.

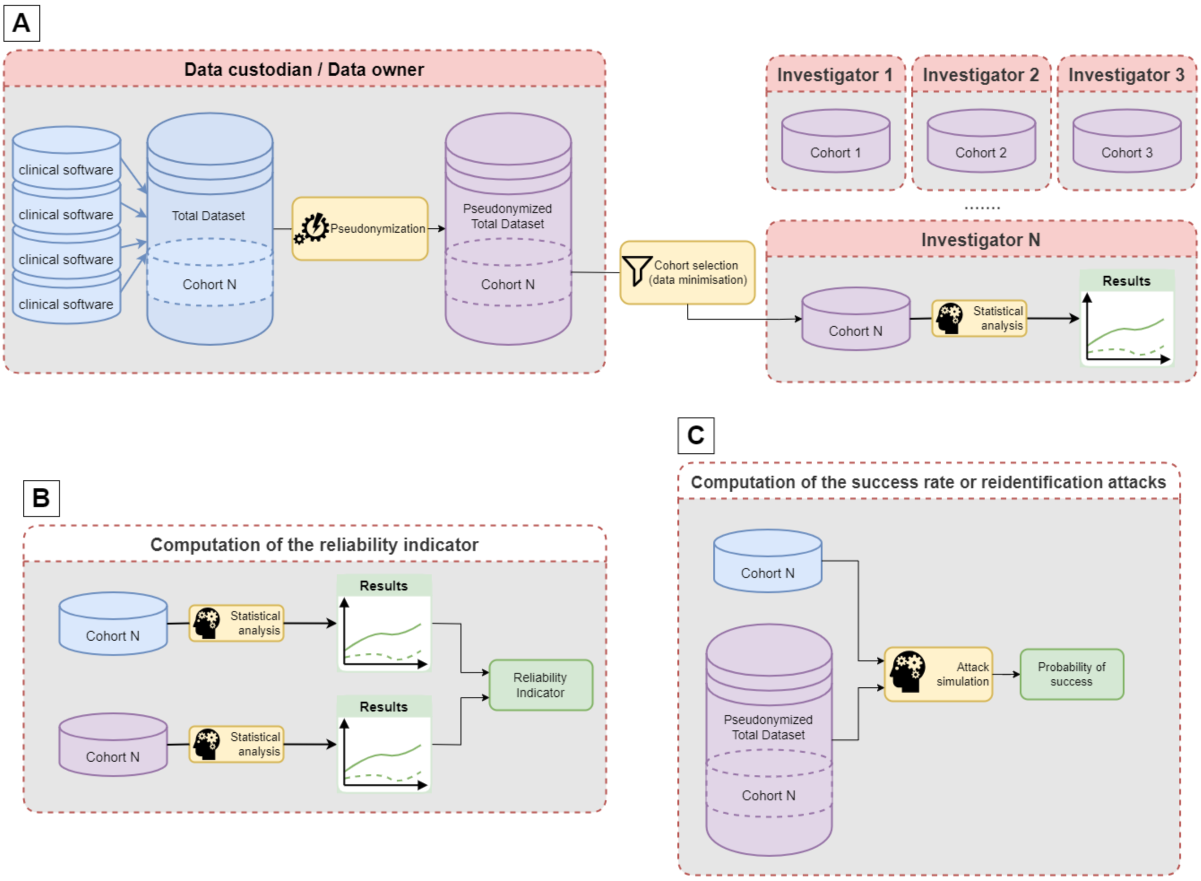

e-Figure 2: Dataflows and processing. A) Delivery of datasets to investigators and final analyses in the context of the AP-HP's clinical data warehouse. Data accesses of data custodians, owners and investigators are shown as dashed boxes. Processing of our study: B) computation of the unreliability indicator and C) the average success rate of reidentification attacks. Not-pseudonymized and pseudonymized data are shown in blue and purple, respectively.

III. Uniqueness computation

A targeted record was not unique among the total dataset if at least two records of the pseudonymized dataset could have resulted from an application of the pseudonymisation scheme on the targeted record, considering in the analysis only the attacker's a priori knowledge (i.e., sex, date of birth, data of death, hospitals, entrance dates, exit dates in most of the analyses). Uniqueness of a record was consequently a random binary variable that depended on the parameters δt drawn at the pseudonymisation stage.

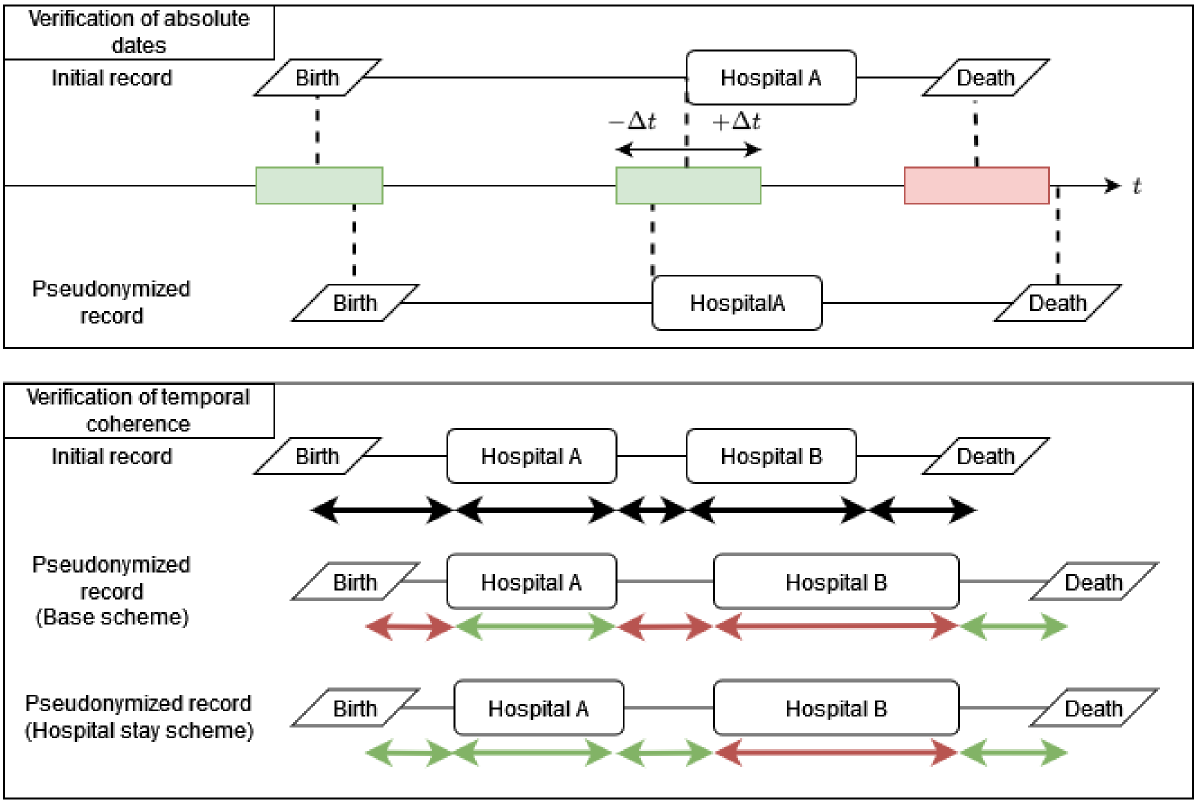

e-Figure 3: Comparison of pseudonymized records with a targeted record to measure uniqueness. A) The birth, death and admission dates of a pseudonymized record are compared to a range of values that depend on the dates of the not-pseudonymized record, the pseudonymisation scheme and its parameterisation. B) The temporal coherence of records is verified comparing durations between events. Depending on the pseudonymisation scheme, the coherence criterion may be more or less stringent. Verified constraints are shown in green and violated ones in red.

A naive estimation of a cohort's average uniqueness would have been computationally demanding as one should have compared each initial record in the cohort to all the records in the total pseudonymized dataset, leading to a quadratic complexity $O(n_{cohort} \times n_{total})$. We adopted two measures to lower this computational overhead. First, we estimated each cohort's uniqueness considering only a random sample of its records. Second, we applied a blocking strategy. Therefore, a sequence of characters starting by the sex (M or F), a chronologically ordered sequence of hospital-related trigrams and a letter indicating whether the patient was dead or alive (D or A) was associated with each record. For instance, a female patient having three successive hospitalisations in hospitals PSL, TNN and HMN and that finally died had the sequence F-PSL-TNN-HMN-D. When the attacker's a priori knowledge does not include hospitalisations, the sequence used is reduced using sex and vital status only. In the case of the Hospital Stay pseudonymisation scheme, as the order of hospitalisations may be altered by

pseudonymisation, we sorted alphabetically the list of visited hospitals. Computational overhead was then reduced by comparing records dates only when they were in the same block, i.e., shared the same sequence. To compare the dates, we took into account the temporal coherence which is either preserved or altered by the pseudonymisation scheme (see e-Figure3).
A random sample of 500 records was used to estimate each measure of privacy assessment (i.e., success rate and uniqueness). Indicators for the total cohort and the cancer cohort were computed with a sample of 100,000 and 10,000 patients, respectively. All analyses were performed using statsmodel 0.13.5 (3). For sequence analyses we used the Traminer v2.2.7 R package (4).

IV. Advanced description of the population

In this section we present the distribution of records among various subgroups whose average uniqueness were computed and additional results.

Considering the entire population cohort, the 3-month rehospitalisation rate was 16% for the base and birth pseudonymisation schemes, regardless of parameterisation. When a stay pseudonymisation with a Δt of 10, 100 and 1000 days applied, the rates were 17%, 15% and 4% respectively. We found a similar effect in the bariatric surgery cohort: 6.2% for the base and birth pseudonymisation schemes, regardless of parameterisation vs. 5.5%, 4.1% and 1.2% when applying a Δt of 10, 100 and 1,000 days respectively for the same stay pseudonymisation schemes.

A)

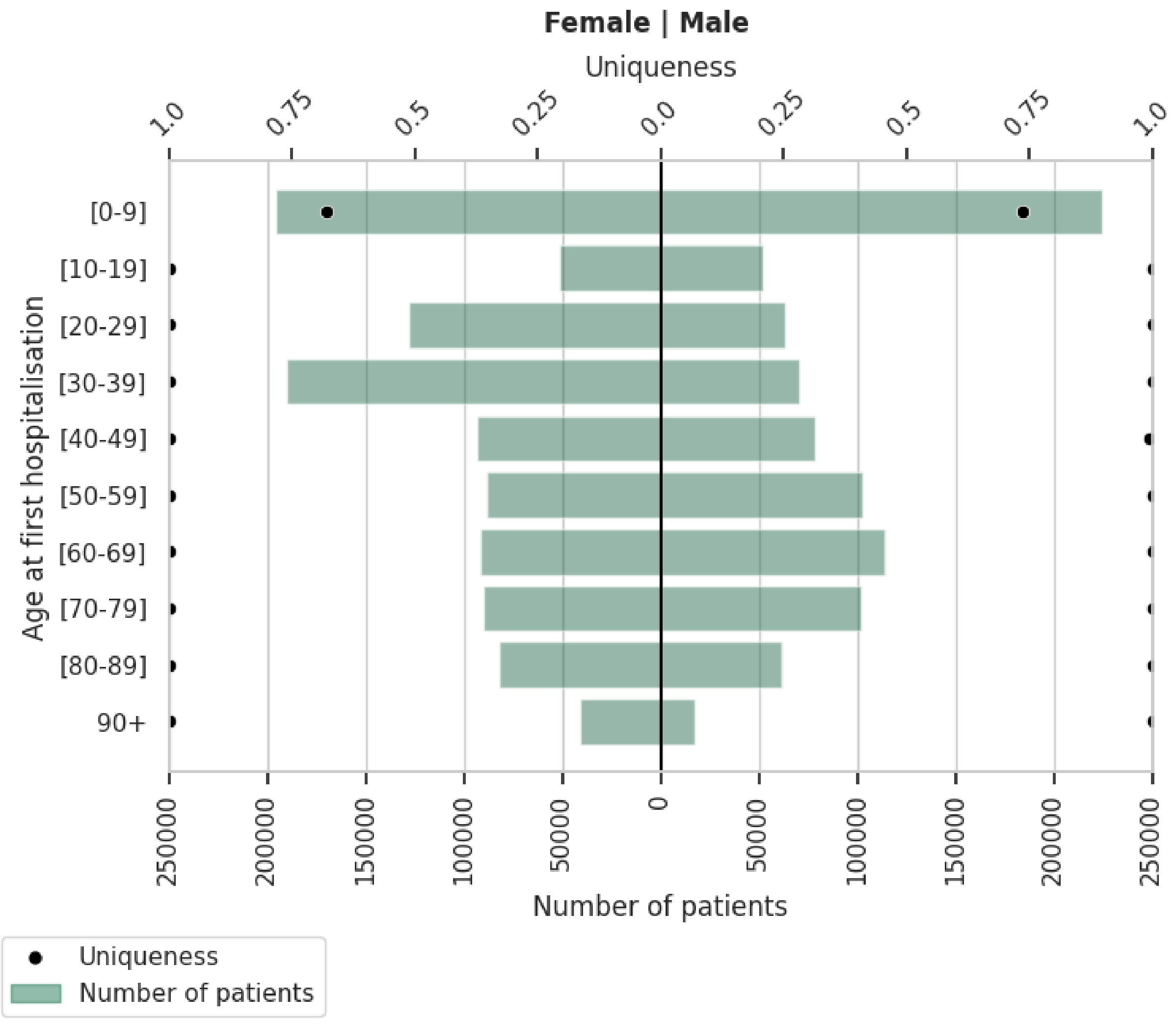

B)

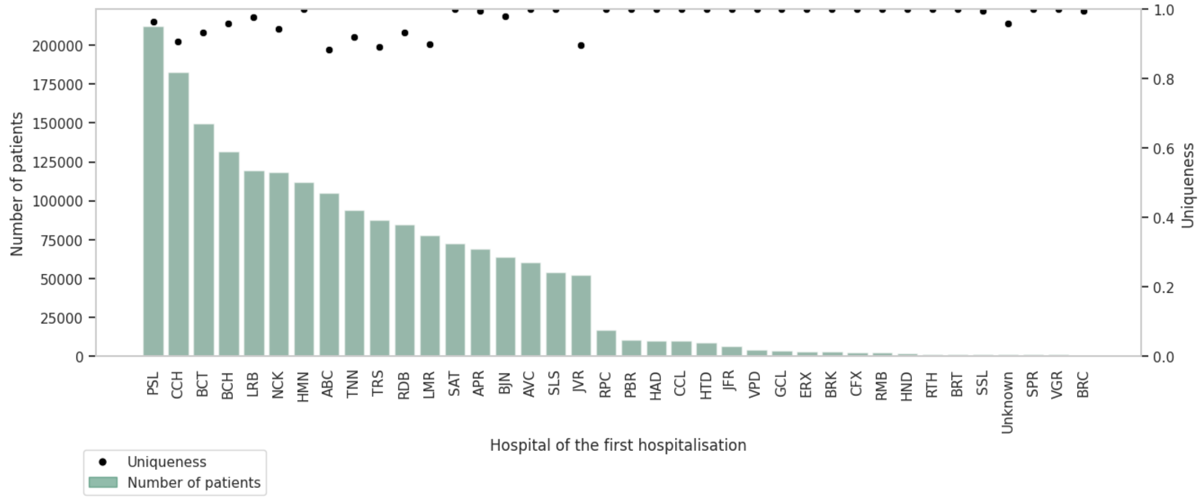

C)

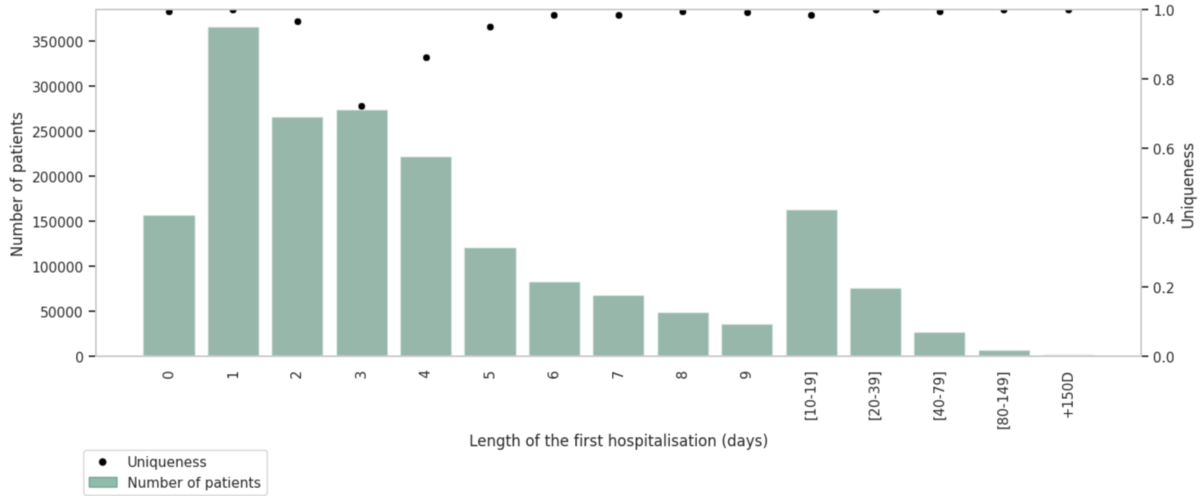

D)

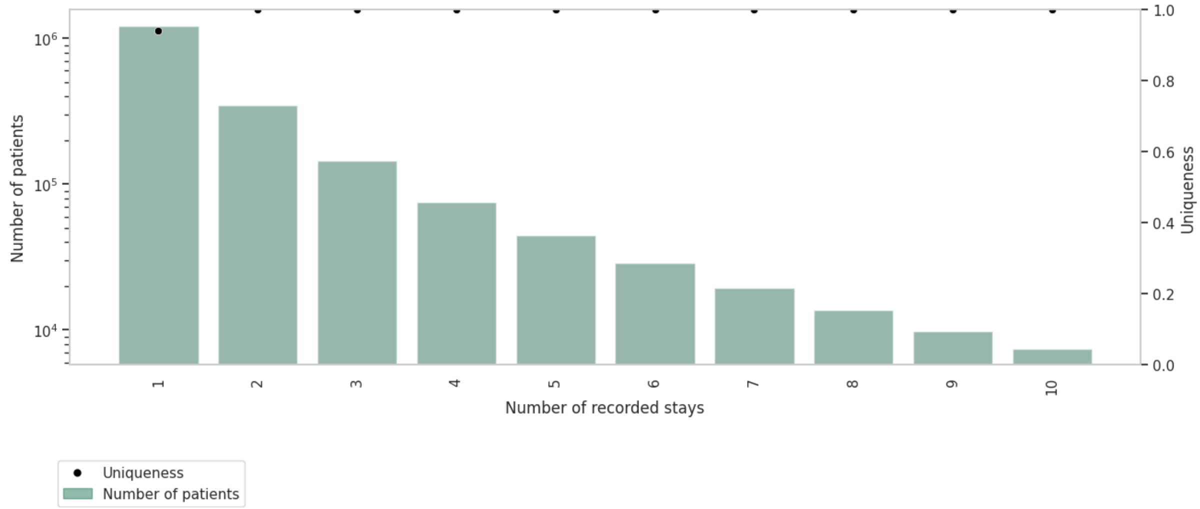

e-Figure 4: Number of records (bars) and average uniqueness (dots) of the overall dataset stratified by A) age at admission of the first record's hospitalisation and sex, B) hospital of the first admission, C) length of the first hospitalisation and D) number of hospitalisations.

Age category		0-14		15-29		30-44		45-59		60-74		75-89		90+	
Pseudonymisation scheme	Shift parameter (days)	Unreliability indicator	Uniqueness	Unreliability indicator	Uniqueness	Unreliability indicator	Uniqueness	Unreliability indicator	Uniqueness	Unreliability indicator	Uniqueness	Unreliability indicator	Uniqueness	Unreliability indicator	Uniqueness
Absence	0	0	0.78	0	1.00	0	1.00	0	1.00	0	1.00	0	1.00	0	1.00
Base	10	0	0.60	0	1.00	0	1.00	0	1.00	0	1.00	0	1.00	0	1.00
	100	0	0.55	0	0.99	0	0.99	0	0.99	0	1.00	0	1.00	0	1.00
	1000	0	0.45	0	0.94	0	0.91	0	0.93	0	0.96	0	0.97	0	1.00
Birth	10	**<0.001**	0.54	**<0.001**	0.96	**<0.001**	0.99	**<0.001**	0.96	**<0.001**	0.98	**<0.001**	0.99	**<0.001**	1.00
	100	**0.001**	0.26	**0.001**	0.50	**0.001**	0.49	**0.001**	0.49	**0.001**	0.69	**0.001**	0.80	**0.001**	0.92
	1000	**0.089**	0.22	**0.066**	0.34	**0.072**	0.34	**0.067**	0.38	**0.067**	0.50	**0.066**	0.56	**0.141**	0.68
Hospital Stay	10	**0.041**	0.54	**0.056**	0.98	**0.047**	0.98	**0.034**	0.97	**0.026**	0.98	**0.063**	0.98	**0.126**	1.00
	100	**0.038**	0.24	**0.011**	0.50	**0.023**	0.48	**0.035**	0.52	**0.046**	0.67	**0.006**	0.78	**0.099**	0.91
	1000	**0.596**	0.12	**0.501**	0.22	**0.512**	0.21	**0.492**	0.24	**0.498**	0.40	**0.482**	0.49	**0.627**	0.56

e-Table 1: Association between privacy and unreliability of results estimated by age category at first stay and three pseudonymisation schemes. The overall cohort was used. Privacy was evaluated through the uniqueness in the dataset given attacker's knowledge and integrity of the study's result was evaluated through an unreliability indicator (see methods section), the lower the better in both cases. For each age category we coloured in green the pseudonymisation schemes that did not impact reliability, i.e., no changes in the research results of each use case scenario. We highlight in bold police the unreliability indicators that are impacted by the pseudonymisation algorithms vs. the absence of it.

V. Target-in-cohort scenario

Cohort		Hospitalisation's characterisation (total dataset)		Seasonal bronchiolitis		Seasonal flu		Bariatric surgery readmission		Pancreatic cancer		Cancer care pathways	
Pseudonymisation scheme	Shift parameter (days)	Unreliability indicator	Uniqueness	Unreliability indicator	Uniqueness	Unreliability indicator	Uniqueness	Unreliability indicator	Uniqueness	Unreliability indicator	Uniqueness	Unreliability indicator	Uniqueness
Absence	0	0	0.94	0	0.99	0	1.00	0	1.00	0	1.00	0	1.00
Base	7	0	0.88	**0.001**	0.98	**0.001**	1.00	0	1.00	**0.001**	1.00	0	1.00
	10	0	0.88	**0.001**	0.98	**0.002**	1.00	0	1.00	**0.001**	1.00	0	1.00
	30	0	0.88	**0.006**	0.96	**0.011**	1.00	0	1.00	**0.004**	1.00	0	1.00
	100	0	0.87	**0.087**	0.93	**0.078**	1.00	0	1.00	**0.010**	1.00	0	1.00
	1000	0	0.82	**0.223**	0.76	**0.257**	1.00	0	0.99	**0.083**	1.00	0	1.00
Birth	7	**<0.001**	0.86	**0.001**	0.87	**0.001**	1.00	0	1.00	**0.001**	1.00	**0.8968**	1.00
	10	**<0.001**	0.86	**0.001**	0.80	**0.002**	1.00	0	1.00	**0.001**	1.00	**0.26157**	1.00
	30	**<0.001**	0.75	**0.006**	0.50	**0.011**	1.00	0	0.99	**0.004**	1.00	**0.89502**	0.98
	100	**<0.001**	0.50	**0.087**	0.29	**0.078**	0.98	0	0.91	**0.011**	1.00	**0.19039**	0.91
	1000	**0.001**	0.38	**0.224**	0.21	**0.257**	0.61	0	0.62	**0.086**	0.97	**0.51601**	0.62
Hospital Stay	7	**0.044**	0.87	**0.001**	0.88	**0.001**	1.00	**0.128**	1.00	**0.026**	1.00	**0.28648**	1.00
	10	**0.047**	0.86	**0.001**	0.80	**0.002**	1.00	**0.138**	1.00	**0.031**	1.00	**0.06584**	1.00
	30	**0.059**	0.74	**0.006**	0.49	**0.011**	1.00	**0.096**	0.99	**0.081**	1.00	**0.68327**	0.99
	100	**0.019**	0.49	**0.083**	0.28	**0.079**	0.98	**0.232**	0.92	**0.089**	1.00	**0.77224**	0.90
	1000	**0.373**	0.28	**0.222**	0.17	**0.255**	0.57	**0.744**	0.49	**0.355**	0.97	**0.48932**	0.52

e-Table 2: Association between privacy (target-in-cohort scenario) and unreliability of results estimated in the case of six archetypal electronic health record studies and three pseudonymisation schemes. Privacy was evaluated through the uniqueness in the dataset given attacker's knowledge and integrity of a study's result was evaluated through an unreliability indicator (see methods section), the lower the better in both cases. For each study we coloured in green the pseudonymisation schemes that did not impact reliability, i.e., no changes in the research results of each use case scenario. We highlight in bold police the unreliability indicators that are impacted by the pseudonymisation algorithms vs. the absence of it.

VI. Success rate – random-target scenario

Cohort		Hospitalisation's characterisation (total dataset)		Seasonal bronchiolitis		Seasonal flu		Bariatric surgery readmission		Pancreatic cancer		Cancer care pathways	
Pseudonymisation scheme	Shift parameter (days)	Unreliability indicator	Success rate	Unreliability indicator	Success rate	Unreliability indicator	Success rate	Unreliability indicator	Success rate	Unreliability indicator	Success rate	Unreliability indicator	Success rate
Absence	0	0	0.94	0	0.012	0	0.009	0	0.003	0	0.002	0	0.095
Base	7	0	0.88	**0.001**	0.012	**0.001**	0.009	0	0.003	**0.001**	0.002	0	0.095
	10	0	0.88	**0.001**	0.012	**0.002**	0.009	0	0.003	**0.001**	0.002	0	0.095
	30	0	0.88	**0.006**	0.012	**0.011**	0.009	0	0.003	**0.004**	0.002	0	0.095
	100	0	0.87	**0.087**	0.012	**0.078**	0.009	0	0.003	**0.010**	0.002	0	0.095
	1000	0	0.82	**0.223**	0.010	**0.257**	0.009	0	0.003	**0.083**	0.002	0	0.094
Birth	7	**<0.001**	0.86	**0.001**	0.011	**0.001**	0.009	0	0.003	**0.001**	0.002	**0.8968**	0.095
	10	**<0.001**	0.86	**0.001**	0.010	**0.002**	0.009	0	0.003	**0.001**	0.002	**0.2616**	0.095
	30	**<0.001**	0.75	**0.006**	0.008	**0.011**	0.009	0	0.003	**0.004**	0.002	**0.895**	0.090
	100	**<0.001**	0.50	**0.087**	0.007	**0.078**	0.008	0	0.002	**0.011**	0.002	**0.1904**	0.080
	1000	**0.001**	0.38	**0.224**	0.007	**0.257**	0.007	0	0.002	**0.086**	0.001	**0.516**	0.069
Hospital Stay	7	**0.044**	0.87	**0.001**	0.011	**0.001**	0.009	**0.128**	0.003	**0.026**	0.002	**0.2865**	0.095
	10	**0.047**	0.86	**0.001**	0.011	**0.002**	0.009	**0.138**	0.003	**0.031**	0.002	**0.0658**	0.095
	30	**0.059**	0.74	**0.006**	0.008	**0.011**	0.009	**0.096**	0.003	**0.081**	0.002	**0.6833**	0.091
	100	**0.019**	0.49	**0.083**	0.006	**0.079**	0.008	**0.232**	0.002	**0.089**	0.002	**0.7722**	0.079
	1000	**0.373**	0.28	**0.222**	0.005	**0.255**	0.007	**0.744**	0.001	**0.355**	0.001	**0.4893**	0.057

e-Table 3: Association between privacy (random-target scenario) and unreliability of results estimated in the case of six archetypal electronic health record studies and three pseudonymisation schemes. Privacy was evaluated through the success rate of re-identification attacks and integrity of a study's result was evaluated through an unreliability indicator (see methods section), the lower the better in both cases. For each study we coloured in green the pseudonymisation schemes that did not impact reliability, i.e., no changes in the research results of each use case scenario. We highlight in bold police the unreliability indicators that are impacted by the pseudonymisation algorithms vs. the absence of it.

VII. References

1. Tannier X, Wajsbürt P, Calliger A, Dura B, Mouchet A, Hilka M, et al. Development and Validation of a Natural Language Processing Algorithm to Pseudonymize Documents in the Context of a Clinical Data Warehouse. Methods Inf Med [Internet]. 2024 Mar; Available from: https://hal.science/hal-04752779

2. Moor M, Banerjee O, Abad ZSH, Krumholz HM, Leskovec J, Topol EJ, et al. Foundation models for generalist medical artificial intelligence. Nature. 2023 Apr 13;616(7956):259–65.

3. Seabold S, Perktold J. statsmodels: Econometric and statistical modeling with python. In: 9th Python in Science Conference. 2010.

4. Gabadinho A, Ritschard G, Müller NS, Studer M. Analyzing and Visualizing State Sequences in *R* with **TraMineR**. J Stat Softw [Internet]. 2011 [cited 2025 Mar 21];40(4). Available from: http://www.jstatsoft.org/v40/i04/

VIII. RECORD's statement checklist

	Item No.	STROBE items	Location in manuscript where items are reported	RECORD items	Location in manuscript where items are reported
Title and abstract					
	1	(a) Indicate the study's design with a commonly used term in the title or the abstract (b) Provide in the abstract an informative and balanced summary of what was done and what was found		RECORD 1.1: The type of data used should be specified in the title or abstract. When possible, the name of the databases used should be included.<br><br>RECORD 1.2: If applicable, the geographic region and timeframe within which the study took place should be reported in the title or abstract.<br><br>RECORD 1.3: If linkage between databases was conducted for the study, this should be clearly stated in the title or abstract.	Title and Abstract<br><br>Abstract<br><br>NA
Introduction					
Background rationale	2	Explain the scientific background and rationale for the investigation being reported			Introduction

Objectives	3	State specific objectives, including any prespecified hypotheses			Introduction
Methods					
Study Design	4	Present key elements of study design early in the paper			Data utility for epidemiological research; Pseudonymisation and minimisation; Attacks
Setting	5	Describe the setting, locations, and relevant dates, including periods of recruitment, exposure, follow-up, and data collection			Study design and population; Data utility for epidemiological research; Details on archetypal EHR studies (appendix)

Participants	6	*(a) Cohort study* - Give the eligibility criteria, and the sources and methods of selection of participants. Describe methods of follow-up<br><br>*Case-control study* - Give the eligibility criteria, and the sources and methods of case ascertainment and control selection. Give the rationale for the choice of cases and controls<br><br>*Cross-sectional study* - Give the eligibility criteria, and the sources and methods of selection of participants<br><br>*(b) Cohort study* - For matched studies, give matching criteria and number of exposed and unexposed<br><br>*Case-control study* - For matched studies, give matching criteria and the number of controls per case		RECORD 6.1: The methods of study population selection (such as codes or algorithms used to identify subjects) should be listed in detail. If this is not possible, an explanation should be provided.<br><br>RECORD 6.2: Any validation studies of the codes or algorithms used to select the population should be referenced. If validation was conducted for this study and not published elsewhere, detailed methods and results should be provided.<br><br>RECORD 6.3: If the study involved linkage of databases, consider use of a flow diagram or other graphical display to demonstrate the data linkage process, including the	Study design and population; Data utility for epidemiological research; Details on archetypal EHR studies (appendix)<br><br>Data utility for epidemiological research<br><br>NA

				number of individuals with linked data at each stage.	

Variables	7	Clearly define all outcomes, exposures, predictors, potential confounders, and effect modifiers. Give diagnostic criteria, if applicable.		RECORD 7.1: A complete list of codes and algorithms used to classify exposures, outcomes, confounders, and effect modifiers should be provided. If these cannot be reported, an explanation should be provided.	Data utility for epidemiological research; Details on archetypal EHR studies (appendix); Outcomes and statistical analysis
Data sources/ measurement	8	For each variable of interest, give sources of data and details of methods of assessment (measurement).<br><br>Describe comparability of assessment methods if there is more than one group			Data utility for epidemiological research; Details on archetypal EHR studies (appendix); Outcomes and statistical analysis
Bias	9	Describe any efforts to address potential sources of bias			Advanced description of the population (appendix)
Study size	10	Explain how the study size was arrived at			Study design and population
Quantitative variables	11	Explain how quantitative variables were handled in the analyses. If applicable, describe which groupings were chosen, and why			Data utility for epidemiological research; Details on archetypal EHR studies (appendix); Outcomes and statistical analysis;

					Uniqueness computation (appendix)

Statistical methods	12	(a) Describe all statistical methods, including those used to control for confounding<br><br>(b) Describe any methods used to examine subgroups and interactions<br><br>(c) Explain how missing data were addressed<br><br>(d) *Cohort study* - If applicable, explain how loss to follow-up was addressed<br><br>*Case-control study* - If applicable, explain how matching of cases and controls was addressed<br><br>*Cross-sectional study* - If applicable, describe analytical methods taking account of sampling strategy<br><br>(e) Describe any sensitivity analyses			Data utility for epidemiological research; Details on archetypal EHR studies (appendix); Outcomes and statistical analysis;

Data access and cleaning methods		..		RECORD 12.1: Authors should describe the extent to which the investigators had access to the database population used to create the study population.<br><br>RECORD 12.2: Authors should provide information on the data cleaning methods used in the study.	Data and code sharing
Linkage		..		RECORD 12.3: State whether the study included person-level, institutional-level, or other data linkage across two or more databases. The methods of linkage and methods of linkage quality evaluation should be provided.	NA
Results					

Participants	13	(a) Report the numbers of individuals at each stage of the study (*e.g.*, numbers potentially eligible, examined for eligibility, confirmed eligible, included in the study, completing follow-up, and analysed)<br><br>(b) Give reasons for non-participation at each stage.<br><br>(c) Consider use of a flow diagram		RECORD 13.1: Describe in detail the selection of the persons included in the study (*i.e.,* study population selection) including filtering based on data quality, data availability and linkage. The selection of included persons can be described in the text and/or by means of the study flow diagram.	Results; Advanced description of the population

Descriptive data	14	(a) Give characteristics of study participants (*e.g.*, demographic, clinical, social) and information on exposures and potential confounders<br><br>(b) Indicate the number of participants with missing data for each variable of interest<br><br>(c) *Cohort study* - summarise follow-up time (*e.g.*, average and total amount)			Advanced description of the population<br><br>Results
Outcome data	15	*Cohort study* - Report numbers of outcome events or summary measures over time<br><br>*Case-control study* - Report numbers in each exposure category, or summary measures of exposure<br><br>*Cross-sectional study* - Report numbers of outcome events or summary measures			NA

Main results	16	(a) Give unadjusted estimates and, if applicable, confounder-adjusted estimates and their precision (e.g., 95% confidence interval). Make clear which confounders were adjusted for and why they were included<br><br>(b) Report category boundaries when continuous variables were categorized<br><br>(c) If relevant, consider translating estimates of relative risk into absolute risk for a meaningful time period			Results
Other analyses	17	Report other analyses done—e.g., analyses of subgroups and interactions, and sensitivity analyses			Advanced description of the population
Discussion					
Key results	18	Summarise key results with reference to study objectives			Discussion

Limitations	19	Discuss limitations of the study, taking into account sources of potential bias or imprecision. Discuss both direction and magnitude of any potential bias		RECORD 19.1: Discuss the implications of using data that were not created or collected to answer the specific research question(s). Include discussion of misclassification bias, unmeasured confounding, missing data, and changing eligibility over time, as they pertain to the study being reported.	Discussion
Interpretation	20	Give a cautious overall interpretation of results considering objectives, limitations, multiplicity of analyses, results from similar studies, and other relevant evidence			Discussion
Generalisability	21	Discuss the generalisability (external validity) of the study results			Discussion
Other Information					

Funding	22	Give the source of funding and the role of the funders for the present study and, if applicable, for the original study on which the present article is based			Fundings
Accessibility of protocol, raw data, and programming code		..		RECORD 22.1: Authors should provide information on how to access any supplemental information such as the study protocol, raw data, or programming code.	Data and code sharing
